# SNP Polymorphisms Are Associated with Environmental Factors in Sockeye Salmon Populations Across the Northwest Pacific: Insights from Redundancy Analysis

**DOI:** 10.3390/genes15111485

**Published:** 2024-11-19

**Authors:** Anastasia M. Khrustaleva

**Affiliations:** Institute of Gene Biology, Russian Academy of Sciences, Vavilova Str., 34/5, 119334 Moscow, Russia; nastia.khrust@gmail.com

**Keywords:** *Oncorhynchus nerka*, sockeye salmon, SNP, adaptive variability, neutral variability, environmental factors, RDA

## Abstract

The SNP variation in sockeye salmon across the Asian part of its range was studied in 23 samples from 16 lake–river systems of the West Pacific Coast to improve understanding of genetic adaptation in response to spawning watersheds conditions. Identification of candidate SNPs and environmental factors that can contribute to local adaptations in sockeye salmon populations was carried out using redundancy analysis (RDA), a powerful tool for landscape genetics proven to be effective in genotype–environment association studies. Climatic and hydrographic indices (7 indices in total), reflecting abiotic conditions in freshwater habitats of sockeye salmon and characterizing the temperature regime in the river basin, its variability during the year, the amount of precipitation, as well as the height of the maximum tide in the estuary, were used as predictor factors. Among the 45 analyzed SNPs, several loci (*ALDOB-135*, *HGFA,* and *RAG3-93*) correlated with predictors gradients along the northwest Pacific coast were identified. The putative candidate loci localized in genes involved in the immune and inflammatory responses, as well as genes encoding temperature-sensitive enzymes and some hormones regulating ion homeostasis in fish during the anadromous migration and smoltification, were potentially associated with environmental conditions in natal rivers. The findings could have implications for aquaculture, conservation, and resource management in the context of global climate change.

## 1. Introduction

Pacific salmon are anadromous fish of the genus *Oncorhynchus* that spend half of their lives rearing in the ocean and return to freshwater to reproduce. Sockeye salmon, *O. nerka* Walbaum, is one of the three most abundant species of Pacific salmon both in Asia and in North America. It is characterized by a long freshwater life span and represented within its range by many isolated stocks [1]. The high fidelity of sockeye salmon to their natal streams allows adaptation to the local environment, resulting in a range of life history characteristics and genetic variation among discrete populations [2].

Environmental factors play a primary role in the occurrence of adaptations in natural populations. The heterogeneity of the environment within the species’ range causes different local selection pressures in different watersheds [3]. The dynamics of sockeye salmon (and the Pacific salmon in general) abundance and productivity are thought to be particularly sensitive to climate variations because their anadromous life cycle exposes them to a variety of climate-related stressors in both marine and freshwater environments [4]. The most critical points for sockeye salmon characterized by long-term freshwater growing periods are both biotic and abiotic conditions in the spawning and rearing watersheds.

The amount of precipitation in the pre-spawning season affects the hydrological river recharge and determines the total area of available spawning grounds. Deficiency of precipitation and groundwater, and hence low water levels, favors a situation when large fish cannot reach spawning grounds or spawn successfully; in addition, in such years, they are more at risk of being eaten by predators in shallow water [5]. With a lack of water, the dependence of fish on the oxygen and CO_2_ concentrations, pH, and temperature in the water also increases [5]. On the other hand, high water flow in high-water years, as well as the complexity and duration of the spawning route, lead to a significant increase in energy consumption during the anadromous migration. Hence, the mortality of spawners will increase, and the probability of eggs getting into the nest will decrease, in addition to the possibility of them being washed out of the nest before or after instillation.

High summer air temperatures and intense water heating often cause heightened mortality in migrating and spawning fish. Upriver migration survival of adults is reduced at water temperatures of >18 °C [4,6,7,8]. The effect of temperature can be either direct and hence associated with the intensification of enzymatic reactions or indirect, for example, leading to a decrease in the concentration of oxygen dissolved in water. According to the concept of oxygen- and capacity-limited thermal tolerance (OCLTT), the oxygen concentration in tissues may decrease due to limitations in the oxygen delivery system capacity at high temperatures and a deficiency of the mitochondrial oxidative capacity at low temperatures [9]. On the other hand, warm temperature (i.e., 15–18 °C, depending on the stock) reduces aerobic scope in sockeye salmon, limiting the fish’s ability to allocate energy to essential tissues during the migration [10,11]. Under extremely high temperatures, aerobic scope is reduced to such an extent that continued migration can lead to anaerobic activity, exhaustion, and death by lactic acidosis or cardiac collapse [12]. At temperatures outside the tolerance range, survival is maintained for a limited time by residual aerobic activity, then by anaerobic metabolism, and finally by molecular defenses by heat shock proteins and antioxidant defenses. Genes coding proteins involved in energy, transport, and other processes during spawning migration up the river are under strong selection pressure since, during this period, energy expenditure and the load on the cardiovascular system increase significantly and must be distributed between the needs of maintaining migratory activity, movement upstream, sexual maturation, and spawning behavior (fish do not feed during this period). In addition, high water temperatures are associated with an increased risk of developing infections and invasions in fish organisms because of increased susceptibility of the immune system to them due to heat stress, activation of pathogenic microorganisms and parasites, increasing their contagiousness and invasiveness [8,13].

It is accepted that the period of embryonic-larval development is the most unfavorable in terms of mortality of Pacific salmon; its rate can reach 77–92% of the number of eggs [1]. The main factors at this time are the hydrological conditions in the nest–water flow, temperature, and oxygen supply [5]. At low winter temperatures and low water content, nests located on small shallow streams can freeze, which leads to complete or partial death of eggs [5]. Moreover, water temperature is critical to egg development and incubation rate [14]. Numerous studies have demonstrated that times of hatching and larval emergence from gravel are inversely related to incubation temperature [14,15,16].

While in the postembryonic period and freshwater feeding, abiotic factors no longer play a key role (for river populations, crisis moments can only be a sharp decrease in water level in rivers and abnormally high temperatures with limited food) [5]; the smoltification, downstream migration, and estuarine period remain the most critical in terms of juvenile survival. This is due to physiological stress associated with the restructuring of the osmoregulatory mechanisms in the body of the fry. Mortality of smolts during this period is highly dependent on the downstream migration conditions and the type of estuary: its length, productivity, specificity, and speed of the water flow, and salinity and temperature of the water, as well as on the age, size, and fitness of juveniles [17]. In addition, climatic conditions during the downstream migration are also important; the “smoltification window” can narrow as the water temperature rises.

The study of adaptive genetic polymorphism and its role in the evolution and formation of the population structure of a species is one of the main fundamental problems of population and ecological genetics. Apparently, having discovered a relationship between the frequency of a certain allele and environmental conditions, i.e., by identifying candidate loci, it is possible to identify indicator genes involved in local adaptations. Understanding the latter is critical to determining how quickly and to what extent particular salmonid populations will respond to habitat deterioration, global climate change, fisheries- or farming-induced evolution, and interactions with hatchery- or captive-reared counterparts [3]. Local adaptation studies are also increasingly important to the definition and application of conservation units within species for legal protection and effective management of salmon stocks. Understanding the current and future impacts of climate change is particularly relevant for commercial fish species such as sockeye salmon, as environmentally driven changes in their productivity can have significant economic, social, and cultural consequences [18].

Studies of local adaptations in North American sockeye salmon populations have been conducted quite intensively over the past 15 years [3,10,19,20,21,22]. However, most of them have focused on identifying the variability underlying the divergence of sockeye salmon ecotypes (beach-spawning and river/stream) and anadromous and resident forms [22,23,24,25,26]. Numerous studies of the SNP loci variability (a panel of 45 loci, later expanded to 96 loci) in sockeye salmon populations from Bristol Bay and the southeast coast of Alaska revealed outlier loci in each of the surveyed watersheds [27,28,29,30,31]. Among them, there were SNPs localized in the genes of the MHC class. II, α subunits of glycoprotein hormones of the pituitary gland, growth hormone, stanniocalcin, transferrin, etc. However, until recently, no attempts were made to determine a correlation between the allelic variants of these loci and specific environmental factors that determine the formation of certain local adaptations. Only in the research by Larson et al. [32], a correlation between the allelic diversity of the MHC locus and the water temperature in the river channel was revealed.

The study of genomic polymorphism by single nucleotide substitutions of Asian sockeye salmon is now in the active phase [33,34]. Using a standard panel of 45 SNP loci, the population structure of this species was analyzed in several regions of the Russian Far East [35,36,37]. In a number of watersheds, SNPs have been identified that have undergone various forms of selection, and hypotheses have been proposed on the possible dependence of the loci variability on the water temperature, estuary geomorphology, etc. However, no in-depth studies have been conducted on the adaptive variability of Asian sockeye salmon. At the same time, such data could have implications for salmon aquaculture, conservation, and resource management in the context of global climate change.

Based on the assumption that some SNPs localized in the structural genes or regulatory sequences potentially evolve under the pressure of one or another form of selection or related effects in the genome (background selection, selective sweep, and genetic hitchhiking), and supposing that it is possible to restore the correlation between their variability and the reproductive conditions of a population that determines selection in corresponding genes, in this work we plan to consider some environmental factors that are presumably responsible for the formation of local adaptations in populations of this species on its Asian range and to trace the relationship between the allele frequencies of candidate SNPs and spatial changes in these factors using redundancy analysis (RDA), a type of asymmetric canonical analysis combining ordination and regression. RDA is a powerful tool for landscape genetics that has proven effective in genotype–environment association studies. As demonstrated by simulations, RDA-based methods are characterized by low false-positive and high true-positive rates when compared to generalized linear models (GLMs) or latent factor mixed models (LFMMs) [38].

## 2. Materials and Methods

### 2.1. Study Area and Sample Collection

**Figure 1:**
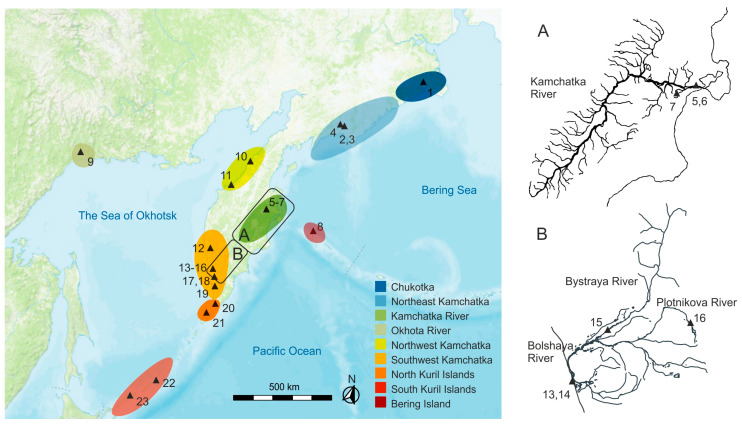
Map of the study area with sampling points (triangles). (**A**) schematic map of Kamchatka River watershed, (**B**) schematic map of Bolshaya River watershed The point’s annotations are given in Table 1. The regions listed in Table 1 are shown in different colors.

Pectoral fin tissue samples were collected over multiple years (2003–2008) in the watersheds of the eastern and western coasts of Kamchatka, Chukotka, mainland coast of the Sea of Okhotsk, Kuril, and Commander Islands (in total, 22 localities from 15 lake–river systems of the Asian coast of the Pacific Ocean) (Table 1, Supplementary Table S1, Figure 1). Sockeye salmon adults were caught using river seine nets in the main rivers and lake creeks at a distance of 5–30 km from the river mouth during the mass run of sockeye salmon, as well as directly in the spawning lakes (Supplementary Table S1). Most Kamchatka samples were obtained from fishing companies immediately after catch in local fisheries. In the Bolshaya River, downstream migrating juveniles (at the stage of the parr-smolt transformation) were caught using minnow seine in the upper reach of Plotnikov River and in the lower course of Bystraya River (Supplementary Table S1). The tissue fragments were fixed in 96% ethanol.

### 2.2. SNP Genotyping

Genomic DNA was extracted with Qiagen DNeasy 96 tissue kits (Qiagen, Valencia, California). TaqMan-PCR using Fluidigm 96.96 Dynamic Arrays (Fluidigm, San Francisco, California) allowed for the genotyping of 95 individuals per 96-well plate (with one inlet used as a no-template control using tris-EDTA buffer) was carried out following the protocol of Seeb et al. [39]. All individuals (*n* = 1226) were genotyped for 45 SNP loci, including three mitochondrial and 42 nuclear loci localized in structural and regulatory genes, dispersed repeats, and EST (Supplementary Table S2). The same panel of 45 SNPs has been previously used in multiple studies of sockeye salmon population structure along the east coast of the North Pacific [27,28,29,30,31,40,41,42], and the present paper extends the scope of the studies to the Asian range. Moreover, the high allele frequency stability for an extended panel of 96 SNPs (including the same 45 loci we used) was revealed for American stocks over 25–42 years (4.9–8.4 generations) [43], which allows us to extrapolate the results obtained for the collection years 2003–2008 to the present or even the future. Of the 45 loci analyzed, only 2 (*One_p53-576* and *One_RAG1-103*) were monomorphic in all the studied samples. Allelic variants of mitochondrial loci were pooled into four combined haplotypes (*One_mtDNA* locus), and the *One_MHC2_190v2* and *One_MHC2_251v2* loci located in the same gene were analyzed separately. Hereinafter, the *One_* prefix in loci names is omitted for brevity.

### 2.3. Climatic and Tidal Data Collection and Analysis

Meteorological data (climate data) from the weather stations located in the lower reaches of the Russian Far East rivers, as well as in the basins of large lake–river systems of the Kamchatka, Chukotka, Okhotsk Region, the Commander and Kuril Islands (Supplementary Figure S1, Supplementary Table S3) for 20 years of observations preceding the sample collection (from 1990 to 2010), otherwise for the entire observation period (from 1950 to 1993) were obtained from the website of the National Oceanic and Atmospheric Administration (NOAA) [44]. From the dataset, a preliminary list of potentially informative environmental factors was selected and tested for cross-correlation, as well as for correlation with the geographic coordinates of the catch sites (Supplementary Figure S2). Among the short list of least correlated climate indices presumable influencing the sockeye salmon generation abundance, determining the differential survival of individuals during the freshwater period, and characterizing the temperature regime of the basin and the amount of precipitation in this area, the following factors were selected as predictors: the average annual air temperature (TAVG–Average Temperature, °C), temperature variation limits (TMAX, TMIN–Maximum, and Minimum temperatures, °C), the duration of the cold (DT00–Number days with minimum temperature ≤ 0 °F) and warm periods (DX70–Number days with maximum temperature > 70 °F) in days, the average annual precipitation amount (PRCP–Precipitation, mm), and the number of rainy/snowy days per year (DP01–Number of days with greater than or equal to 0.1 inch of precipitation) (Table 1, Supplementary Table S4). The annual mean values of each factor were averaged over the entire observation period. The missing data (for West Kamchatka populations of the Palana, Tigil, and Opala rivers) were predicted by a linear approximation of the corresponding factor average value by the latitude of the river basin since for the western coast of Kamchatka, significant and high correlation coefficients were found between all selected indices and the geographic coordinates of the weather station (TAVG: *r* = −0.96 ***, TMIN: *r* = −0.94 ***, TMAX: *r* = −0.88 ***, DT00: *r* = 0.96 ***, DX70: *r* = 0.8 ***, PRCP: *r* = −0.88 ***, DP01: *r* = −0.87 ***, for nine West Kamchatka stations (Supplementary Figure S1)). In order to reduce the correlation of predictors instead of two indices of extreme temperature values (TMIN and TMAX), the value of the air temperature variation range, TLIM (calculated as TMAX–TMIN), was used (Supplementary Table S4).

The estuaries of the studied rivers were classified according to the degree of influence of sea tides, as in [45,46]: tidal/microtidal (tidal height (Δ*H*) is up to 2 m), semi-macrotidal (2 m < Δ*H* < 4 m), macrotidal (Δ*H* ≥ 4 m), and hypertidal (Δ*H* ≥ 6 m). The height of the maximal tide in the corresponding estuary was taken as a predictor (TIDE), and the data from [46], as well as from [47,48], were used. To detail the relationship between the estuary type and the genetic characteristics of the samples, Dr. K. Habicht’s data [31] on the frequencies of 45 SNP loci in a sample from Tigil River (*n =* 107) were used (Supplementary Figure S3, Supplementary Table S5).

### 2.4. Statistical Analysis

To characterize the trends in genetic diversity along the Western Pacific coast, observed and expected heterozygosity (*Ho*, *He*) for each locus (excluding mtDNA SNPs), mean allele count (*n_a_*), and estimates of allelic richness (*Ar*) by rarefaction using the smallest sample size were obtained in hierfstat [49]. To test the correlation between genetic indices and some geographical and climatic features of the spawning watersheds and to visualize the correlation matrices, the stats R package [50] and corrplot R library [51] were used. To describe the sockeye salmon population structure and analyze environmental factors relationships, the principal component analysis (PCA) was performed using the R libraries factoextra [52] and FactoMineR [53]. To select a set of putative neutral loci, a panel of 45 SNP markers was iteratively screened to identify outlier loci at different spatial scales, either analyzing all population samples together or evaluating various combinations of samples from different locations. Outlier detection was performed using coalescent simulations under the hierarchical island model to obtain *p*-values of the locus-specific F-statistic conditioned on observed levels of heterozygosities implemented in Arlequin 3.5 [54]. Twelve loci (*MHC2_190v2*, *MHC2_251v2*, *GPH-414, serpin*, *HGFA* (*p* < 0.01) and *GHII-2165*, *HpaI-99*, *U401-224*, *STC-410*, *ALDOB-135*, *GPDH*, *and Prl2* (*p* < 0.05)) out of forty nuclear polymorphic SNPs were identified as candidates for the directional selection and two (*U504-141* and *LEI-87* (*p* < 0.01)) for the balancing selection (Supplementary Figure S4).

**Figure 2:**
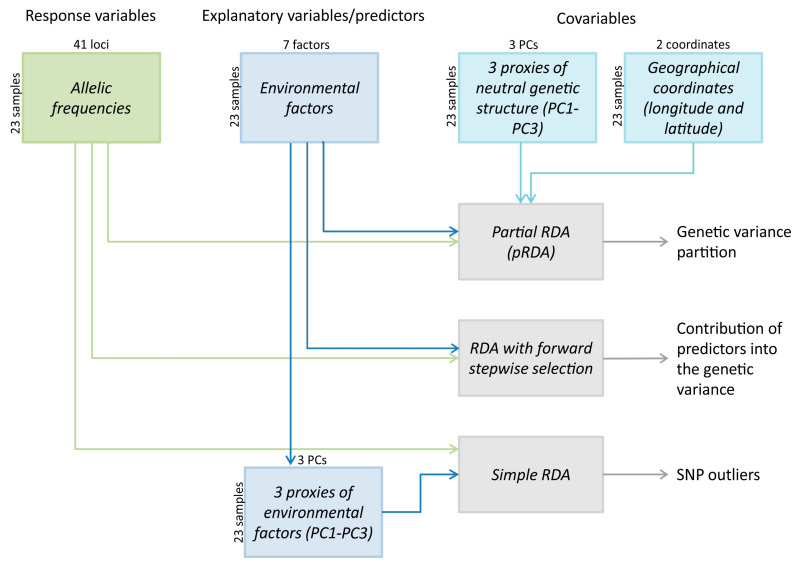
Flowchart of redundancy analysis steps: partial RDA, RDA with forward stepwise selection, simple RDA, and variables used in each step.

In order to identify whether environmental variables could explain variations in allele frequencies among locations, a redundancy analysis (RDA) was conducted (Figure 2) in the vegan R package [55]. Here, RDA with forward stepwise selection was used to identify the relative contribution of each environmental variable on the allelic frequency variation with the Akaike information criterion as described in [56]. The empty model (intercept only) was sequentially complexified by adding explanatory variables one by one, using two stopping criteria to prevent overestimation of the explained variance: a permutation-based significance test (*p*-values were estimated based on 1000 permutations) and the adjusted *R*^2^ of the global model. The goal of the procedure was to maximize the genetic variance explained by a set of environmental predictors. In addition, partial RDA (pRDA) was applied for variance partitioning to decompose the contribution of climate, neutral population structure, and geography in explaining genetic variation. Population allele frequencies were used as the response variable in four different models with four sets of predictor variables: (1) all predictors, (2) seven climate/environment variables, (3) three proxies of neutral genetic structure (population scores along the first three axes of a genetic PCA (PC1-PC3) conducted on the 26 putative neutral loci), and (4) population coordinates (longitude and latitude) to characterize geographical variation.

Finally, a classical RDA procedure was performed. The RDA models linear relationships among the genetic markers and explanatory variables and ordinates this variation along orthogonal axes that condense patterns of covariation between genetic markers and environmental predictors [56]. Each genetic marker will have a score or ‘loading’ along each of the newly built RDA axes. The loci putatively under selection are the markers showing extreme loadings along one or multiple axes. Outliers (loci potentially involved in adaptation to climate/environment) were identified as SNPs with the greatest squared loadings along the RDA axes (i.e., those in the 5% tail of normal distribution or differing from the mean by more than two standard deviations). Thus, a locus that falls outside the 2σ interval can be considered a statistically significant outlier with a confidence level of 95%.

## 3. Results

### 3.1. Differentiation of Asian Sockeye Salmon Populations

**Figure 3:**
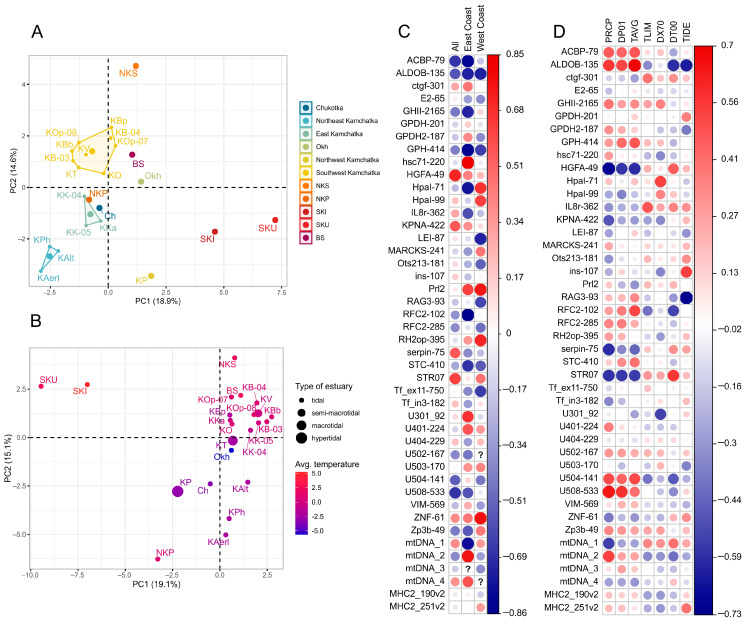
PCA results showing population clustering based on (**A**) allele frequencies of 26 putative neutral SNPs (regions are marked with different colors; some populations are combined into polygons according to the region, and some are outliers), (**B**) allele frequencies of 41 polymorphic SNPs (populations are scaled chromatically by the average temperature, °C, and in size by the type of estuary/the high of tides). Matrices of correlation coefficients between allele frequencies of 41 SNPs and (**C**) the latitude of the river mouth along the all-Asian coast of the North Pacific, the East Cost (Chukotka and East Kamchatka), and the West Coast (Continental coast of the Sea of Okhotsk and West Kamchatka); (**D**) climatic and hydrographic indices reflecting abiotic conditions in the reproductive watershed.

According to the PCA results for 26 potentially neutral loci (Figure 3A), in the space of the first two principal components (explaining in sum 33.5% of dispersion), the samples were clustered mainly according to their belonging to the reproduction region, with the exception of the Palana sample and island populations. When all 41 polymorphic loci were included in the analysis (PC1 and PC2 in sum explain 34.2% of dispersion) (Figure 3B), the samples from the northern populations were concentrated in the center of the cluster of points, while the samples from warmer watersheds were located on the periphery.

### 3.2. Correlation of Genetic Indices with the Latitude and Environmental Factors

To test the relationship between the allele frequencies of 41 polymorphic SNPs and the latitude of the spawning watershed, a correlation analysis was used (Figure 3C). Its results showed a weak (0.20–0.39 [57]) to moderate (0.40–0.59 [57]) correlation of most loci allele frequencies with latitude across the Asian range, only for the *HGFA* locus, a highly significant relationship with the watershed location was observed (*r* = 0.72 ***). However, if we trace the dependence of allelic frequencies on latitude along the northern coast of the Sea of Okhotsk and Western Kamchatka (excluding island populations), high correlation coefficients were revealed for ten loci, and along the Pacific coast (coast of Chukotka and Eastern Kamchatka) for eight loci (Supplementary Table S6).

To test the relationship between the allelic frequencies of 41 SNPs and the environmental factors along the all-Asian coast of the North Pacific, a correlation analysis was carried out (Figure 3D). As can be seen from the correlation matrix, only four values of the Pearson correlation coefficient exceeded the threshold adopted for a high relationship, equal to 0.7; the allele frequencies of the *ALDOB-135* were correlated with the average annual temperature (*r* = 0.7 ***), the allele frequencies of the *RAG3-93* correlated with the estuary type (*r* = −0.73 ***), and the allele frequencies of the *HGFA* correlated with the annual amount of precipitation in the reproduction region (*r* = −0.71 ***). However, many loci, including *ACBP-79*, *ALDOB-135*, *GHII-2461*, *GPH-414*, *HGFA*, *HpaI-436*, *IL8r-362*, *KPNA-422*, *mtDNA*, *pIns-107*, *RAG3-93*, *RF-112*, *serpin*, *STR07*, *U301-92*, *U504-141*, and *U508-533*, showed significant to moderate levels (*r* up to 0.59) of correlation with one or more environmental factors (Supplementary Figure S5).

Intrapopulation genetic diversity estimates across the Asian range slightly increased from south to north (*H_e_*: *r* = 0.608 **, *H_o_*: *r* = 0.518 *, *n_a_*: *r* = 0.423 *, *Ar*: *r* = 0.558 **). However, this trend is mainly determined by populations of northeastern Kamchatka and Chukotka, where both allelic richness and *H_e_* were extremely high (see Table 1), on the one hand, and by samples from the Kuril Islands, where the lowest intrapopulation diversity was revealed, on the other. If we consider the coasts of the Sea of Okhotsk and the Pacific Ocean separately (excluding island populations), then in the first case, a negative correlation between the diversity indices and geographic latitude was discovered (*H_e_*: *r* = −0.415, *H_o_*: *r* = −0.452, *n_a_*: *r* = −0.743 *, *Ar*: *r* = −0.886 ***), while in the second, a positive one (*H_e_*: *r* = 0.228, *H_o_*: *r* = 0.535, *n_a_*: *r* = 0.540, *Ar*: *r* = 0.707). The same trends in the diversity indices distribution along both coasts of the Kamchatka Peninsula remain even if Dr. K. Habicht’s dataset is included in the analysis (Supplementary Figure S6).

### 3.3. Environmental Factors Analysis

**Figure 4:**
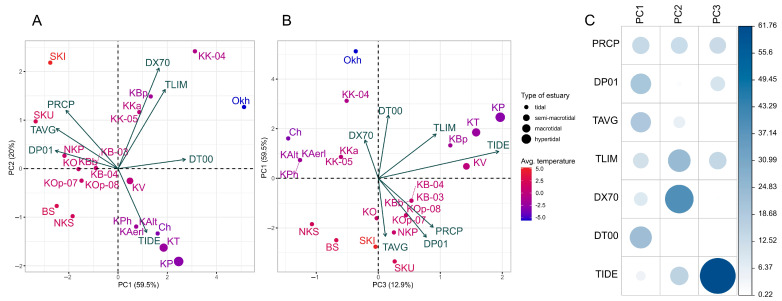
(**A**,**B**) PCA results of climate and hydrographic indices in sockeye salmon reproduction watersheds of the Asian Pacific coast. Three main components characterizing the climate in general (PC1), the degree of climate continentality (PC2), and the type of estuary (PC3) were defined. (**C**) Loading matrix for each ecological variable to PC1–PC3.

The relationships between climatic and hydrographic parameters (ecological factors) were studied using PCA. Three main components were identified, explaining in sum 92.4% of the total variability of environmental variables (Figure 4A,B). The samples were distributed in the space of the first two principal components in accordance with the latitudinal gradient of average temperatures and the degree of contrast of the climatic conditions of the nursing watershed (Figure 4A). When adding the third component, as expected, the horizontal gradation of the populations according to the type of estuary was clearly pronounced (Figure 4B). Such factors as average annual temperature, number of cold and rainy/snowy days per year, and the annual amount of precipitation had the largest loadings (contribution) to the first component (PC1); the temperature variation limits and the number of warm days during a year to the second component (PC2), while the type of estuary to the third component (PC3) (Figure 4C). Thus, it can be generalized that the first component characterizes climatic conditions in general (temperature and precipitation), the third, the type of estuary, and the second one, the variability of air temperature and the number of warm days during the year, i.e., the degree of climate continentality, since inland regions are characterized by more significant annual amplitude of air temperatures and warmer summer, compared to the sea coast.

### 3.4. Relationship Between Environmental Factors and Genetic Features

A forward selection with RDA was used to identify the main ecological variables associated with genetic variation. Four of the seven climate variables were selected (Table 2) by the criteria of variable significance of *p* < 0.05, showing a strong influence of air temperature and precipitation.

By the results of pRDA-based variance partitioning altogether, climate, neutral genetic structure, and geography explained 82.3% of the total genetic variance across Asian sockeye salmon populations (Table 3). The effect of climate was highly significant and explained 54.5% of total genetic variation (66.2% of the variation explained by the full model), suggesting a strong association between genetic variation and environmental gradients (Isolation By Environment, IBE). The pure effect of neutral genetic structure accounted for 17.6% of total genetic variance (21.4% of explained variation), while geographical coordinates accounted for slightly more than 10% (12.4% of explained variation).

To test the hypothesis about the relationship between Asian sockeye salmon genetic structure and ecological factors, we conducted a canonical redundancy analysis (RDA) using the three previously identified principal components (PC1–PC3) associated with the variability of environmental factors as explanatory variables (predictors) and the allele frequencies of 41 SNPs as response variables. According to the RDA results, a fraction of genetic variability explained by environmental factors was 10%, i.e., most of the variability of genetic traits remained unexplained. The first constrained axis (RDA1) explains most of the potentially explainable variability.

A high correlation between the genetic structure and environmental predictors (*r*_RDA1_ = 0.88, *r*_RDA2_ = 0.87, *r*_RDA3_ = 0.81) was typical for all three constrained components. The relationship between the genetic structure and the environment, according to the results of the permutation ANOVA test, was significant (*p* = 0.008 ***). At the same time, the genetic structure changes significantly only along the first principal axis (RDA1: *p* = 0.008 **). The following principal components significantly affect the genetic structure of the sockeye salmon populations of the Asian Pacific coast (the marginal ANOVA): PC1 (*p* = 0.014 *) and PC3 (*p* = 0.028 *).

**Figure 5:**
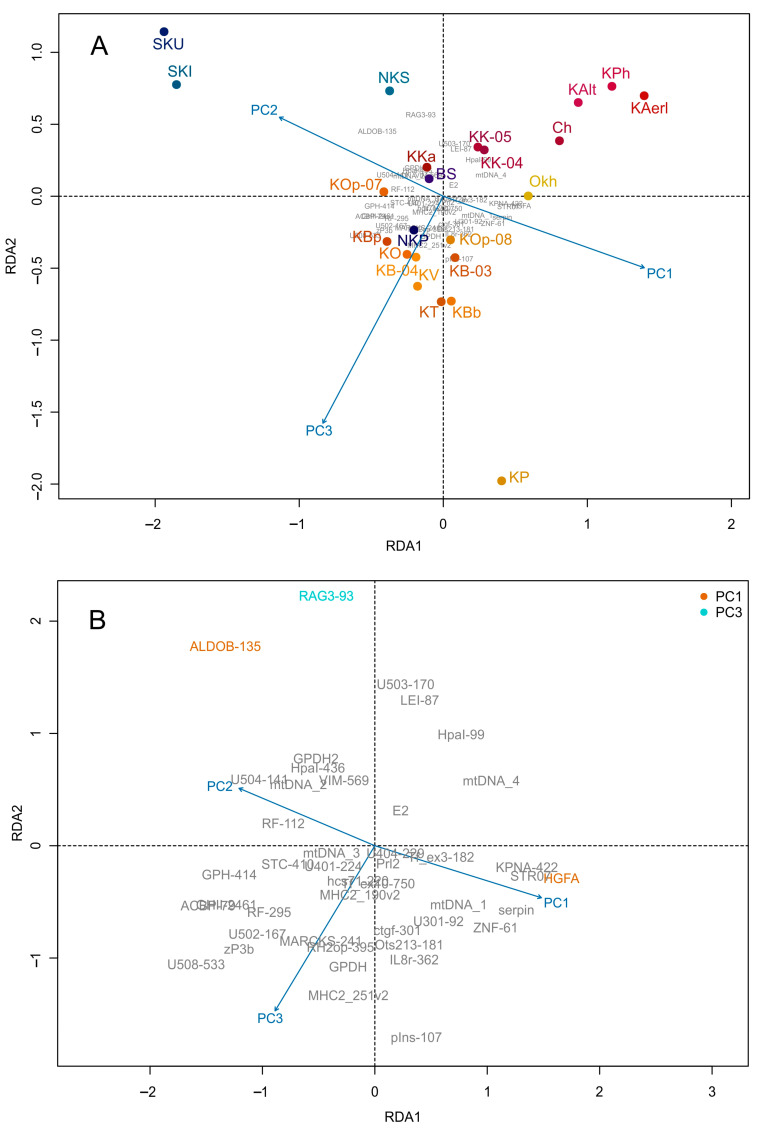
(**A**) RDA triplot displaying predictor factors (3 principal components obtained by PCA analysis of climate and hydrographic indices (PC1–PC3)) (vectors), samples (named points) and dependent variables (SNP loci), and (**B**) biplot for genetic traits (loci correlated with the corresponding predictors are marked in corresponding color).

On the RDA triplot, in the space of the first two constrained components (Figure 5A), one can see which environmental factors most determine the similarity of the samples; the first axis (RDA1) was associated mainly with climatic variables (PC1 and PC2), while the second (RDA2) with the type of estuary (PC3). The projection of each sample point onto the predictor vector reflects its ecological optimum with respect to a given environmental factor. Thus, along the PC1 and PC2 vectors, the points were distributed more or less in accordance with the latitudinal gradient of climatic factors. The southernmost and northernmost of the populations studied were at the opposite poles, along the orthogonal PC3 vector, more or less in agreement with the magnitude of the maximum tide in the corresponding estuary. Along the first constrained axis, samples from the water bodies of the South Kuril Islands were isolated, and along the second, from the Palana River.

The identification of candidate loci for local selection was performed by analyzing the distributions of locus loadings along the corresponding component; only those loci whose loadings deviated from the mean by at least two standard deviations (2σ) were selected. A not-too-conservative threshold was chosen (no more than 95% of loci were rejected) since it was important for us to identify as many potential candidates for analysis as possible. In total, three loci were selected; two of them (ALDOB-135 and HGFA) correlated with the climatic component PC1 and one (RAG3-93) with the estuary type. All candidate loci are marked with color on the RDA biplot (Figure 5B).

Using all the climate and hydrographic indices as predictors and the same RDA algorithm, it was possible to identify eight candidate loci; half of them (*ALDOB-135*, *GPH-414*, *Prl2*, *STC-410*) correlated with the average annual temperature, two (*LEI-87*, *RAG3-93*) were associated with the estuary type, and two (*HGFA* and *U301-92*) with the amount of precipitation and the number of warm days per year, respectively (Supplementary Figure S7). Most of the listed loci, with the exception of *RAG3-93* and *U301-92*, were identified as outliers or candidates for directional/balancing selection. The relationship between the genetic structure and the environment, according to the results of the permutation ANOVA test, was highly significant (*p* = 0.001 ***); the proportion of dispersion explained by environmental factors was 23%. Moreover, the genetic structure changes significantly along the first two axes (RDA1: *p* = 0.004 **, RDA2: *p* = 0.009 **). The ANOVA test showed that factors such as annual precipitation (*p* = 0.001 ***), temperature variation range (*p* = 0.006 **), and number of warm days per year (*p* = 0.029 *), as well as estuary type (*p* = 0.031 *) were significant predictors of allele frequencies in sockeye salmon populations.

## 4. Discussion

The main subject of our pilot study of adaptive polymorphisms in Asian sockeye salmon was to identify, in the SNP panel widely used in genetics studies of American and Asian stocks, potentially selectively loaded SNP loci that may be directly or indirectly (as a result of background selection, selective sweep, or the hitchhiking effect) involved in local adaptations to the reproduction conditions of populations, and key environmental candidate factors associated with such adaptive variability throughout the Asian part of the species range. For this purpose, we attempted to identify the gradient character of polymorphisms of some loci along the northwestern coast of the Pacific Ocean, taking into account its relief and zonality. Assuming that clinal variability of allele frequencies, as well as genetic diversity indices, both in the range as a whole and along the Sea of Okhotsk and Pacific coasts, may be due to both demographic (gene migration, IBD) or historical processes associated with the sockeye salmon range formation in Asia (postglacial expansion, secondary contact), and the latitudinal gradient of various abiotic and biotic factors, our objectives included identifying clines associated with regular changes in climatic and physical geographical conditions and the local adaptations to their gradations.

### 4.1. Population Divergence and Neutral Diversity Trends

As is generally accepted, isolation by distance is often the main driver of intraspecific genetic structure. However, the results of the RDA variance partitioning pure geography model were not significant, so the latitudinal variance was not the defining factor of population divergence. This suggests that there are more complex patterns of genetic variation distribution in sockeye salmon across its range. Trends in intrapopulation diversity are important for identifying such patterns that are often not related to the differential survival of individuals. According to the “abundant center-periphery” hypothesis, the diversity indices usually correlate with the total population size [58,59]. Central populations have the highest effective population size (*Ne*) and gene flow (*m*), reflecting high values of intrapopulation variability and low interpopulation differentiation. Meanwhile, toward the edge of a range, genetic diversity decreases, and the degree of population divergence increases. The decrease in diversity in populations of the northern coast of the Sea of Okhotsk (the Palana and Okhota rivers) and in Kuril Islands populations, located on the subperiphery of the Asian part of the sockeye salmon range, is entirely consistent with this hypothesis. However, this trend was not observed along the Pacific coast of Kamchatka and Chukotka; a decrease in gene diversity was not observed in the northernmost sockeye salmon populations of the Asian part of the range. On the contrary, estimates of expected and observed heterozygosity and allelic richness in the corresponding samples were maximally high. Genetic diversity, in theory, should be higher in populations that have existed for a long time in the territory of former refugia due to the accumulation of mutant alleles. At the same time, theoretically, neutral genetic diversity should decrease with distance from the reproduction watersheds of hypothetical ancestral populations because the colonization of new territories by a species is carried out sequentially with small and less polymorphic groups of individuals. Thus, the result is a gradient of genetic diversity, namely its decrease in the direction of the putative paleodispersive flows. At the same time, the mixing of populations originating from different refugia also leads to an increase in genetic diversity in the secondary contact zone [60]. The highest estimates of *H_o_* and allelic diversity were noted in samples from the Kamchatka River basin (the Dvukhyurtnaya and Elovka rivers) and in a sample from the Pakhachka River. In the Dvukhyurtochnaya R. (a tributary of Elovka R.), we previously revealed extremely high haplotype and nucleotide diversity based on the analysis of the mtDNA control region sequence [61]. Following these findings, we expected the existence of a large refugium in the Kamchatka R. basin during the Last Glacial Maximum (LGM). Both types of data indicate that the expansion of sockeye salmon along the western Pacific coast after the LGM occurred from several centers, one of which was the paleobasin in the middle reaches of the Kamchatka R.; the other one was Beringia (a large refugium located in Alaska, occupying most of the Yukon River basin, the Bering Strait, and the northern part of the Bering Sea, which were dry land at that time due to sea regression). For the Pakhach River population (and other populations of northeastern Kamchatka), on the contrary, a secondary contact scenario with the North American sockeye salmon after the LGM seems more likely [33].

Opposite trends in the diversity indices distribution in the western and eastern Kamchatka populations indicate that the expansion of sockeye salmon during the Holocene transgression after the LGM could have come from different refugia. While the colonization of the western coast of Kamchatka apparently happened sequentially from south to north [61,62]. On the eastern coast, it followed a more complex scenario. Knowledge of the demographic history and ideas about the neutral population structure of the species in the Asian part of its range will allow us to more correctly interpret the correlations between the environmental factors and the genetic divergence of populations.

### 4.2. Patterns of Environmental Factors Change Along the Asian-Pacific Coast

The hydrological regime of most Kamchatka rivers is determined mainly by climatic conditions [63]. In the distribution of climate indicators along the Asian coast of the Pacific Ocean, certain patterns can be traced, caused by its length, geographical location, the influence of surrounding seas and oceans, the movement of air masses, and relief. From our observations, highly reliable negative correlations were found between the geographic latitude of the watershed location and the average annual temperature (*r* = −0.85 ***), as well as the annual amount of precipitation (*r* = −0.91 ***) (at high latitudes, on average, less precipitation is recorded than at more southern ones). According to the PCA results, both indices correlated with each other and characterized the climate as a whole, while other related variables, air temperature variability and the number of warm days during the year, were associated with the degree of climate continentality. These patterns are manifested due to the large length of the coastline in the meridional direction and the landscape zonality of the area of distribution of sockeye salmon on the Asian coast of the Pacific Ocean. The annual amount of precipitation decreases in the direction from the southeast of the range to the northwest from 1000–1300 mm to 300–400 mm [63]. This is due to the fact that the southern territories (the end of the Kamchatka Peninsula and the Kuril Islands) experience a more significant oceanic influence than the northern regions located on the continental coast or in the central part of the peninsula. Thus, for central Kamchatka as a whole, a continental climate with cold winters and warm summers is characteristic, and on the coasts and in the southern part near Cape Lopatka, a maritime climate with relatively mild winters and cool summers [64]. The climate in the upper and middle parts of the Kamchatka River basin, compared with the climate in other regions, deviates to the greatest extent towards the sharply continental [63].

### 4.3. Environmental Variables Potentially Driving Local Adaptation and Candidate Loci Affected by Them

Identification of environmental factors that may affect selective agents and contribute to the local adaptations in sockeye salmon populations was conducted using RDA. Proxy factor PC1 (explaining 59.5% of dispersion), combining such climate characteristics as temperature and precipitation regime (variables PRCP, TAVG, DP01, DT00), was determined to be the most important selective agent in the system since this factor explained the largest share of variance in genetic variables in RDA and had the highest estimates of the correlation coefficient with outlier markers. Moreover, the variables PRCP, TAVG, DP01, and DT00 had the biggest contribution to the allelic frequency variation by the results of the forward selection of individual predictors. Based on the results of RDA analysis, three outlier loci were identified (*ALDOB-135*, *HGFA,* and *RAG3-93*); the variability of the first two were associated with the climatic variable PC1, and the last one, with the type of estuary (component PC3, 12.9% of dispersion).

Numerous studies have shown that temperature is a major factor contributing to local adaptation in sockeye salmon and other salmonids and that growth, metabolism, and immune-related functions may be the main targets of local selection [4,10,21,25,32,65,66,67]. Temperature can influence the growth and survival of fish at all life stages. Ambient temperature significantly affects the intensity of energy metabolism and oxygen consumption in poikilothermic animals, the catalytic activity of enzymes [68,69], and therefore largely determines adaptation processes in a heterogeneous environment. In sockeye salmon, adaptation to temperature causes differences in the timing of the run, spawning, development, and growth of young fish in different types of spawning grounds and different climatic zones, mortality, and seasonal-ecological race formation. In addition, throughout the entire sockeye salmon range, a clear trend towards dependence of the size and weight of spawners on the geographical latitude of the river mouth is observed; the sizes of individuals increase in more northern latitudes [70]. This pattern is caused by the relationship between growth and maturation rates in poikilothermic animals with temperature; the growth rate rises with increasing temperature, but at the same time, the period of growth before maturity is reduced (because the growth rate is rising somewhat slower than the development rate). Consequently, the sizes of adult individuals are reduced [71]. In addition, in more northern populations, an average longer lifespan of individuals is observed due to a higher proportion of fish aged 6 years (age classes 3.3+ and 2.4+). Most often, the cause for this fact is that in the northern lakes with shorter vegetation seasons, juveniles do not have time to gain weight and size and have to remain in the lakes for a longer time. Moreover, the relationship between biological indices (body length and weight) in sockeye salmon of Eastern Kamchatka and latitude is significantly higher than that in fish of Western Kamchatka [70]. These trends can obviously be due to both epigenetic mechanisms and genetic adaptation to different temperature conditions in reproduction watersheds and, apparently, in marine feeding areas as well. At the same time, the contribution and the ratio of both components responsible for such phenotypic variability are quite difficult to estimate. Genetic mechanisms of adaptation, in this case, are explained by the counter-gradient theory [72], according to which low temperatures and a short growing season in northern latitudes contribute to adaptations associated with more efficient plastic and energy metabolism and growth.

Based on the results of RDA and correlation analysis, it was revealed that the polymorphism in the *ALDOB-135* site was associated with climatic conditions, particularly with the average annual water temperature in the native watershed. The *ALDOB-135* substitution is localized in the gene encoding aldolase B, an enzyme that plays an important role in the mechanism of anaerobic digestion of carbohydrates in animal tissues (glycolysis). It is present in all animal cells, but its content is especially high in tissues with intensive carbohydrate metabolism (striated muscles, myocardium, liver). Despite the fact that this locus is located in the intron of the *ALDOB* gene, it can be linked to neighboring exons encoding the functional domains of the enzyme or be in the area of hitchhiking effect with neighboring adaptive genes. For this locus, coldwater (A) and warmwater (G) alleles can be identified with a more or less high degree of certainty. Enzymes may have isoforms (allelic variants) differing in the catalytic activity. The clinal allozyme variability across the range of a species, which does not coincide with the supposed patterns of its paleo dispersal, can be explained by the different adaptability of isoforms to the population’s living conditions. Thus, for sockeye salmon of the North American Pacific coast, clinal variability was shown for the allozyme loci Ldh-B1 and Pgm-1 when the frequencies of relatively rare alleles increased to the northwest [73,74,75]. Currently, eight allozyme loci have been identified in sockeye salmon of North America, the allele frequencies of which correlated with the geographic latitude of the mouths of spawning rivers; in Asian sockeye salmon, there were four such loci [76].

According to the RDA, with the inclusion of all predictor results, the frequencies of the *GPH-414*, *Prl2,* and *STC-410* loci also correlated with temperature. The first one is located in the intron of the *GPH* gene encoding the α-subunit of glycoprotein hormones of the pituitary gland, which perform a wide range of functions in fish, including regulation of growth processes, sexual maturation, formation of spawning coloring, and resistance to high temperatures [77]. The relationship of its variability on such an extended coastline in the meridional direction with the temperature factor is quite expected since the rate of metabolic processes in poikilothermic animals, as well as the duration of embryonic development, growth, and sexual maturation rates, swimming, and feeding activity strongly depend on the temperature conditions of their habitat, and can be interpreted from the standpoint of the counter-gradient theory.

For sockeye salmon as a relatively stenothermic psychrophilous species, the temperature conditions of anadromous migration are critical in terms of survival. The high level of migration mortality may be caused by a combination of temperature-mediated factors. Firstly, an increase in water temperature leads to a decrease in the concentration of dissolved oxygen, the need for which increases due to the increase in energy expenditure on swimming against the current. Second, high water temperatures result in the rise of energy consumption in fish, and migration ceases when energy reserves fall below a critical level [78]. Third, exposure to high water temperatures increases the rate of development of malconditions and infections in fish due to physiological stress and decreased swimming performance [8,13,79,80]. Finally, temperatures close to the upper tolerance limit (i.e., 15–18 °C, depending on the stock) result in a significant reduction in the aerobic capacity (scope) of fish, limiting tissue energy input during migration [10,11]. At extreme water temperatures, the aerobic range is reduced to such an extent that continued migration can lead to anaerobic overload, exhaustion, and death from lactic acidosis or cardiac collapse [12]. Variability in survival responses to temperature, both between populations and among individuals within a population, likely reflects local adaptations to the thermal conditions in which their ancestors reproduced [4,15,81]. As a result, fish from populations adapted to more challenging migratory environments have greater aerobic scope, larger hearts, and better coronary supply. The role of temperature as a major driver of the adaptive divergence of populations has been demonstrated in a number of large-scale landscape genomics studies of North American salmonids [10,82,83,84,85].

*Prl2* and *STC-410* substitutions were found in genes whose products are involved in the regulation of ion balance in fish organisms, which is especially important during the transition from seawater to freshwater and vice versa. The first locus is located in the coding sequence of the prolactin gene, in the third position of the codon. Prolactin is the main hormone that adapts the organism of anadromous fish to fresh water. It increases plasma osmolarity in fresh water, enhances the retention of sodium ions and reduces their leakage, increases the concentration of sodium and chlorine ions in the blood, has a hypercalcemic effect, reduces the water permeability of osmoregulatory organs due to the effect on the activity of the gills (in particular, by changing the type and number of gill chloride cells), kidneys, intestines, skin, and bladder in bony fish [86]. The prolactin gene was actively expressed in sexually mature sockeye salmon approaching the river mouth during physiological restructuring of the body before anadromous migration [87]. It was experimentally proven that the expression of prolactin receptors decreased in downstream chinook salmon during acclimation to seawater, and increased expression of this gene was associated with high mortality of catadromous juveniles [88,89]. The *STC-410* substitution is localized in the intron of the stanniocalcin gene. In fish, this is a classic glycoprotein hormone secreted by the corpuscles of Stannius, inhibiting calcium absorption through the gills and intestines in response to hypercalcemia [90]. In addition, it stimulates the absorption of inorganic phosphorus (phosphates) by renal tubule cells, i.e., it is responsible for calcium and phosphorus homeostasis [91]. Stanniocalcin is involved in fish adaptation to hyperosmotic conditions in salt water. Thus, these are antagonist hormones that regulate ion homeostasis in fish during anadromous migration. Moreover, smoltification and downstream migration are considered the most critical periods of the life cycle of Pacific salmon in terms of mortality, along with anadromous migration. The relationship between the efficiency of osmoregulation during catadromous migration and water temperature has been examined in a number of studies on Atlantic salmon and rainbow trout [92,93,94]. A direct dependence of the success of adaptation to seawater on the size of downstream migrants and its negative correlation with the water temperature is observed. The negative impact of hyperthermia on the survival of juveniles during the transition to seawater may be due to the sharp increase in cortisol levels in response to both stimuli (high temperature and salinity), as well as its effect on the activity of a number of enzymes, including the temperature-dependent Na/K-ATPase [92].

Not only fry migration and smoltification but also the estuarine period, ending with the entrance of smolts into the marine environment, are critical in the life of Pacific salmon in terms of the juvenile’s survival. During this period, when significant natural mortality is associated with stress due to adaptation to the marine environment, juveniles experience the most significant selection pressure. It is generally accepted that the formation of the generations’ abundance of anadromous fish reproducing in the rivers of Kamchatka is associated with hydrology and morphology of estuaries of various types [46].

Estuaries provide a number of functions for Pacific salmon, including an osmoregulatory gradient and migration corridor for smolts [17]. It has been established that their importance increases significantly in systems with limited freshwater feeding opportunities and in years of high salmon density [95]. Due to the hydrological and morphological features of various types of Kamchatka estuaries, certain factors that determine the survival of juveniles predominate in each of them. In the lagoon-channel estuaries (rivers of the southwestern and eastern coasts of Kamchatka), such a factor is high variability in water salinity; in the lagoon–lake estuaries (on the eastern coast), it is low dissolved oxygen concentration and high concentrations of H_2_S, and in channel estuaries (northwest), it is significant tidal fluctuations in water level. The high tides on the northeastern coast of the Sea of Okhotsk have a significant impact on the hydrological regime of the estuaries located at its apex. The most extreme conditions for juveniles are observed in the hypertidal estuaries because of the stress caused by significant fluctuations in water level, salinity, temperature, turbidity, speed, and the current direction, etc. [46]. The Palana River estuary can be classified as hypertidal, i.e., it is characterized by significant spatio-temporal variability of all abiotic factors (primarily the level, speed, and direction of water currents, salinity, temperature, turbidity) under the influence of hyperhigh (more than 10 m) tides. The tidal intrusion of brackish waters into the river bed can reach several kilometers and cause a strong reverse current, to overcome which juveniles have to spend more energy; in turn, high turbidity of water in the estuarine zone and active mixing of the water mass hinder the visual and olfactory orientation and search for food of smolts. Thus, the conditions of fry migration in Palana River can be classified as extreme; the survival of juveniles in the estuarine period can be achieved only by high swimming activity and endurance of smolts, as well as a high degree of readiness for the transition to salt water (completion of pre-catadromous transformation, reaching a certain body size, corresponding physiological changes). According to the concept of a “smoltification window,” the survival of juveniles is largely determined by the timing of sea migration; pre-smolts that have not completed the parr-smolt transformation or de-smolts that have remained in freshwater too long and have reverted to a physiology more suited to freshwater, have significantly reduced survival [88]. In the conditions of a hypertidal estuary, juveniles can linger in the desalinated intrusion zone for several days, which will complicate their subsequent adaptation to high salinity and will result in an increase in mortality. Obviously, the elimination of fingerlings (0+) and yearlings (1+) in such conditions will be especially high.

Loci associated with tidal height are located in genes whose products are involved in the regulation of innate immune response, inflammation and cellular homeostasis (*LEI-87*), and the formation of immunoglobulin diversity and T-cell receptors (*RAG3-93*). In the work of Larson et al. [96], devoted to the search for associations and identification of QTL determining heat tolerance, body condition index, length, and body weight in sockeye salmon, out of 45 SNPs used in this study, 27 were analyzed, and only *RAG3-93* was included in the QTL group associated with the quantitative trait such as body length, and annotated as involved in metabolism and biosynthesis. Apparently, sockeye salmon populations’ divergence by these genes involved in the formation and regulation of innate and specific immunity is associated with local differences in historical disease rates among smolts in response to physiological stress due to the difficulty of forcing the tidal estuarine zone of their natal rivers. As is known, the complex physiological transformation during the process of smoltification requires large energy costs, which leads to a weakening of the immune system [97]. Recent transcriptomics analyses have demonstrated repressed expression of genes associated with the immune system during smoltification and after seawater transfer [98,99].

The role of precipitation and temperature as selective forces has also been demonstrated in other salmonids [10,82,83,84] as well as in other taxa [100]. In anadromous salmon, Micheletti et al. [84] suggested that extremely high precipitation may result in additional energy costs during migration to spawning grounds. Conversely, low water combined with high temperatures may complicate access to spawning grounds and cause strong selective pressures [101]. In our experiment, the variability of the *HGFA-49* locus, located in the hepatocyte growth factor (HGF) gene, encoding a glycoprotein involved in organogenesis, tissue repair, and regeneration of the liver, also acting as a growth factor for a wide range of tissues and cell types, was associated with the climate variable (PC1) and average annual precipitation amount. Activated after hepatocyte damage, HGF induces angiogenesis, stimulates cell proliferation and migration, inhibits Fas-induced apoptosis and slows down the development of fibrosis after inflammation, stimulates the proliferation of some types of epithelial cells, as well as vascular endothelial cells and melanocytes [102]. The GO categories for this gene in Danio rerio also include differentiation and migration of CNS neurons, cerebellar morphogenesis, negative regulation of the apoptotic process, positive regulation of protein phosphorylation, and proteolysis. It seems difficult to link these functions with the characteristics of river outflow and its effect on the survival of sockeye salmon spawners; however, its participation in the development of the coronary circulation system can be assumed. However, upon closer analysis, it was found that the correlation of the minor allele frequency with the river latitude characteristic for this locus was found for the entire data set (including island populations) but not for the western and eastern coasts separately. In addition, *HGFA-49* and *serpin* turned out to be outlier loci responsible for the divergence of the populations of the South Kuril Islands (maximum *F_CT_*); their frequencies in these two samples were inverted relative to the frequencies in the other samples of the dataset. Based on these observations, we consider it important to approach its selection as a candidate locus with caution.

The frequency of the anonymous locus *U-301* correlated with the number of warm days per year. The sequence containing this SNP was identified as a fragment of the FK506-binding protein 12 gene (*FKBP12*). The protein encoded by this gene is a member of the immunophilin protein family, which plays a role in immunoregulation and the main cellular processes associated with the folding and transfer of proteins. Since the number of warm days per year characterizes the duration and severity of the hot summer period, it is also associated with the temperature conditions of the spawning run of sockeye salmon, in particular its late (summer) form and the conditions in the spawning grounds of both forms. As noted above, temperature is a major predictor of mortality before spawning (i.e., pre-spawn mortality or inability to mate), while the association between high temperature and pre-spawn mortality most often being due to high disease prevalence [13,103]. Transcriptome analysis of Fraser River sockeye salmon runners migrating to spawning grounds showed that fish were more vulnerable to temperature-induced disease and premature mortality when migrating in warmer conditions (at elevated but not close to lethal water temperatures) [66].

## 5. Conclusions

Thus, it was demonstrated that on a large geographic scale (the entire Asian part of the range) among a limited panel of markers, we were able to identify putative candidate SNPs that can be directly or indirectly involved in local adaptations in sockeye salmon populations. Single nucleotide substitutions localized in genes whose products are involved in the regulation or performing of the immune and inflammatory response in the fish organism were more often than others associated with local temperature or other hydrological conditions in natal rivers. The impact of climatic and hydrographic factors that go beyond the optimal values causes stress reactions in the fish organism, weakening its immune defense and leading to differential infection and subsequent elimination of the least adapted individuals. In addition, genes encoding temperature-sensitive enzymes and some hormones, including those that regulate ion homeostasis in fish during anadromous migration and smoltification, are also thought to evolve under selection pressure responsible for the genetic divergence of Asian sockeye salmon populations.

Although the results obtained in this study require future verification on a wider panel of loci and a larger area, they suggest the existence of relationships between environmental factors and potentially adaptive genetic variability in sockeye salmon, as well as the contribution of local conditions in spawning watersheds to the evolutionary processes that shape the genetic population’s structure of this species in the Asian part of its range. Clinal changes in the allele frequencies of some candidate loci make it possible to identify the most adapted genotype/allele in certain conditions (for example, cold and warm alleles of the *ALDOB-135* locus), which, if their adaptability is reliably confirmed empirically, could potentially allow these data to be used in Pacific salmon aquaculture and sustainable fisheries management in a changing climate.

## Figures and Tables

**Table 1:** Sampling locations, population IDs, date of catch, genetic diversity estimates for 41 SNP loci (*He*—mean expected heterozygosity, *Ar*—allelic richness), and climatic and hydrographic conditions in the spawning watersheds: PRCP—precipitation, mm; TAVG—average temperature, °C; DX70—number days with maximum temperature > 70 °F; DT00—number days with minimum temperature ≤ 0 °F; DP01—number of days with greater than or equal to 0.1 inches of precipitation; TLIM—air temperature variation range, °C; TIDE—height of the maximal tide, m. In brackets, standard deviation (SD). *—data from Habicht et al. [31].

Samples Characteristics							Genetic Diversity		Climatic and Hydrographic Characteristics of Sampling Sites						
#	Region	Location	Pop ID	Coordinates	Date of Catch	*n*	*Ar (SD)*	*H_e_ (SD)*	*PRCP (SD)*	*TAVG* (*SD*)	*DX70* (*SD*)	*DT00* (*SD*)	*DP01* (*SD*)	*TLIM* (*SD*)	*TIDE*
1	Chukotka	Vaamochka Lake	Ch	62.54° N, 176.81° E	July 2004	50	1.8(0.55)	0.26(0.2)	395.09(152.1)	−3.4(0.88)	3.17(1.59)	76.25(13.86)	122.75(20.22)	5.87(0.49)	1.5
2	Kamchatka, North–East	Apuka River, early run	KAerl	60.42° N, 169.85° E	June 2008	18	1.82(0.53)	0.26(0.19)	467.35(113.59)	−1.95(1.13)	0.77(0.91)	54.5(17.22)	134.25(20.69)	5.73(0.39)	1.4
3		Apuka River, late run	KAlt	60.42° N, 169.85° E	June 2008	28	1.87(0.49)	0.25(0.18)	467.35(113.59)	−1.95(1.13)	0.77(0.91)	54.5(17.22)	134.25(20.69)	5.73(0.39)	1.4
4		Pakhacha River	KPh	60.8° N, 169.07° E	June 2005	59	1.86(0.46)	0.27(0.19)	467.35(113.59)	−1.95(1.13)	0.77(0.91)	54.5(17.22)	134.25(20.69)	5.73(0.39)	1.4
5	Kamchatka, East coast	Kamchatka River, late run	KK–04	56.19° N, 162° E	June–July 2004	82	1.78(0.43)	0.25(0.19)	421.51(80.16)	−0.82(0.89)	53.3(9.27)	89.85(16.81)	125.3(16.15)	12.84(0.81)	1.5
6		Kamchatka River, early run	KK–05	56.19° N, 162° E	June 2005	15	1.74(0.58)	0.27(0.22)	638.48(106.72)	0.21(0.85)	33.52(9.81)	59(13.2)	151.19(13.63)	8.21(0.48)	1.5
7		Azabachje Lake, Bushuyka River	KKa	56.12° N, 161.85° E	July 2004	81	1.76(0.42)	0.25(0.19)	638.48(106.72)	0.21(0.85)	33.52(9.81)	59(13.2)	151.19(13.63)	8.21(0.48)	1.5
8	Commander Islands	Bering Island, Sarannoye Lake	BS	55.27° N, 166.15° E	August 2007	58	1.63(0.58)	0.18(0.2)	668.08(114.57)	2.79(0.63)	0.06(0.24)	0(0)	211.44(14.21)	3.21(0.14)	1.5
9	Continental coast of the Sea of Okhotsk	Okhota River	Okh	59.47° N, 142.95° E	July 2004	80	1.68(0.47)	0.24(0.21)	423.97(108.86)	−5.67(0.65)	45.39(8.81)	141.28(8.81)	86.72(12.33)	12.62(0.75)	2.8
10	Kamchatka, North–West	Palana River	KP	59.04° N, 160.21° E	July 2003	94	1.73(0.45)	0.25(0.19)	483.65	−3.01	8.22	86.01	137.09	7.93	8
11		Tigil River *	KT	58° N, 158.27° E	June 2002	107	1.81(0.41)	0.17(0.18)	523.83	−2.26	6.98	75.23	144.04	7.68	7
12	Kamchatka, South–West coast	Bolshaya Vorovskaya River	KV	54.36° N, 156.1° E	July 2007	45	1.8(0.51)	0.24(0.2)	777.12(107.32)	0.06(0.75)	5.61(2.5)	57.44(12.27)	163.06(14.43)	9.11(0.42)	4.5
13		Bolshaya River	KB–03	52.63° N, 156.26° E	July 2003	91	1.82(0.5)	0.25(0.19)	811.72(129.81)	0.95(0.7)	2.75(1.94)	33.7(10.54)	191.4(17.61)	7.64(0.29)	2.2
14		Bolshaya River	KB–04	52.63° N, 156.26° E	August 2004	90	1.83(0.49)	0.26(0.2)	811.72(129.81)	0.95(0.7)	2.75(1.94)	33.7(10.54)	191.4(17.61)	7.64(0.29)	2.2
15		Bolshaya River drainage, Bistraya River	KBb	52.92° N, 156.61° E	July–August 2004	33	1.79(0.53)	0.25(0.21)	811.72(129.81)	0.95(0.7)	2.75(1.94)	33.7(10.54)	191.4(17.61)	7.64(0.29)	2.2
16		Bolshaya River drainage, Plotnikova River	KBp	53.1° N, 157.76° E	August 2004	39	1.79(0.53)	0.26(0.21)	896.35(189.83)	−1.77(0.82)	20.37(7.75)	89.42(14.71)	175.95(16.47)	12.05(0.81)	2.2
17		Opala River	KOp–07	52.13° N, 156.48° E	July 2007	50	1.77(0.45)	0.23(0.19)	746.26	1.89	0.1	15.54	182.51	5.91	2.1
18		Opala River	KOp–08	52.13° N, 156.48° E	July–August 2008	31	1.83(0.5)	0.25(0.2)	746.26	1.89	0.1	15.54	182.51	5.91	2.1
19		Ozernaya River	KO	51.48° N, 156.57° E	August 2003	95	1.82(0.48)	0.25(0.2)	744.43(165.85)	1.27	0.87	9.91(6.63)	207.33(18.98)	7	2
20	North Kuril Islands	Shumshu Island, Bettobu Lake	NKS	50.75° N, 156.26° E	August 2008	50	1.71(0.55)	0.22(0.21)	679.79(166.1)	2.11(0.49)	0(0)	0(0)	167.36(23.67)	3.13(0.36)	1.9
21		Paramushir Island, Glukhoye Lake	NKP	50.49° N, 155.85° E	July 2008	48	1.67(0.53)	0.17(0.17)	1103.28(263.36)	1.1(0)	0	2.43(5.56)	192.88(25.27)	6.2(0)	1.9
22	South Kuril Islands	Urup Island, Tokotan Lake	SKU	45.86° N, 149.8° E	July–August 2008	35	1.58(0.58)	0.18(0.21)	1269.63(187.02)	2.5(0.14)	2(1)	0.22(0.67)	229.08(16.67)	5.6(0.14)	1
23		Iturup Island, Krasivoye Lake	SKI	44.62° N, 147.21° E	October 2006	50	1.55(0.61)	0.17(0.21)	1179.89(166.65)	5.23(0.57)	25.57(10.89)	1.29(3.45)	199.33(14.11)	6.63(0.35)	1

**Table 2:** Climatic variables were identified as significantly associated with genetic variation using forward variable selection with RDA. *AIC*—Akaike information criterion, *F* and *p*-value—F-test results, ′—*p* < 0.1, *—*p* < 0.05, **—*p* < 0.01.

#	Climate Variable	Variable ID	*AIC*	*F*	*p*-Value
1	Precipitation, mm	PRCP	208.8	3.7318	0.005 **
2	Average temperature, °C	TAVG	209.84	2.636	0.020 *
3	Number of days with ≥0.1 inch of precipitation	DP01	210.39	2.0785	0.035 *
4	Number of days with minimum temperature ≤ 0.0 °F (−17.8 °C)	DT00	210.71	1.7582	0.085 ′
5	Number of days with maximum temperature > 70 °F (21.1 °C)	DX70	211.06	1.4223	0.13
6	Height of the maximal tide, m	TIDE	211.14	1.3362	0.21
7	Temperature variation range, °C	TLIM	211.26	1.2265	0.24

**Table 3:** The influence of climate, geography, and neutral genetic structure on genetic variation decomposed with pRDA. The proportion of explainable variance represents the total constrained variation explained by the full model. *R*^2^—coefficient of determination, *Var*—variance, ***—*p* < 0.001.

Model	*R* ^2^	*Var*	*p*-Value	% of Explainable Variance	% of Total Variance
Full model	0.823	7461.1	0.001 ***	100	
Pure climate model	0.545	4938.3	0.001 ***	66.2	54.5
Pure neutral population structure model	0.176	1596	0.001 ***	21.4	17.6
Pure geography model	0.102	926.8	0.053	12.4	10.2
Residual (unexplained) variance		1605.7			17.7
Total variance		9066.8			100

## Data Availability

The datasets generated and analyzed during the current study are available from the author upon reasonable request.

## Supplementary Materials

The following supporting information can be downloaded at: https://www.mdpi.com/article/10.3390/genes15111485/s1, Figure S1. The NOAA weather station location in the studied area. Figure S2. A scheme for predictor selection for the analysis of correlation between environmental factors and allele frequencies of 41 polymorphic SNP loci in sockeye salmon: selection of predictors–removal of predictors with high cross-correlation estimates–addition of new parameters (2–3 iterations)−calculation of derived parameters. Figure S3. Schematic map of the study area with sampling points (triangles–our collection, red circles–data from Habicht et al.). The point’s annotations are given in Tables 1 and S5. The regions listed in Table 1 are shown in different colors. Figure S4. Examples of output images for outlier-SNP detection tests using Arlequin 3.5 for four combinations of samples: (A)–all samples, (B)–all samples vs. KP sample, (C)–all continental samples vs. all island populations, (D)–all samples vs. BS sample. Loci falling above 5% (in the upper part of the graph) and below 1% (in the lower part of the graph) quantile limits were removed as outliers. Here serpin, HGFA, GPH-414, MHC2_190v2, STC-410, ALDOB-135, GPDH, and U504-141 and LEI-87 are considered as outliers. Figure S5. Matrix of the correlation coefficients between allele frequencies of 41 SNPs and environmental indices reflecting abiotic conditions in the reproductive watershed. The numbers in the cells are the correlation coefficients. Figure S6. Expected heterozygosity (He) and allelic richness (Ar) for 41 SNP loci as a function of latitude for sockeye salmon from the Asian coast of the Pacific Ocean: East Coast–East coast of Kamchatka and Chukotka, West Coast–West coast of Kamchatka and the Okhota River. The entire dataset of Habicht et al. (Table S5) was included in the analysis. Figure S7. (A)−RDA triplot displaying predictor factors (seven climate and hydrographic indices) (vectors), samples (named points), and dependent variables (SNP loci), and (B)−biplot for genetic traits (B) (loci correlated with the corresponding predictors are marked in corresponding color). Table S1. Sampling regions and locations, nursery lakes in the watershed, population IDs, and sampling methodology: date and place of catch, coordinates of a river mouth or a lake of catch, fishing gear, and collector name if known. Table S2: Characteristics of 45 SNP loci. He–mean expected heterozygosity, Ho–mean observed heterozygosity, na–mean allele count per locus, FST–fixation index by locus. Table S3. The NOAA weather station data used in this study included weather station location, years of observation, number of observations, and corresponding spawning lake–river systems. Table S4. The environmental factors used in the present study. Table S5. Samples characteristics, regions and locations, population IDs, date of catch, coordinates, and summary statistics for 40 SNP loci according to the data from (Habicht et al., 2010): mean expected (He) heterozygosities, allelic richness (Ar). Table S6. Correlation coefficients of allele frequencies of SNP loci with the latitude of the spawning watersheds for which the correlation was significant in at least one test (***—*p* < 0.001, **—*p* < 0.01, *—*p* < 0.05) along the all Asian coast of the North Pacific (All), coast of Chukotka and East Kamchatka (East Cost), and the Continental coast of the Sea of Okhotsk and West Kamchatka (West Coast).
