# Supplementary material for: SNP Polymorphisms Are Associated with Environmental Factors in Sockeye Salmon Populations Across the Northwest Pacific: Insights from Redundancy Analysis
Source: Genes (Basel). 2024 Nov 19;15(11):1485. doi: 10.3390/genes15111485 (PMC11593481; doi:10.3390/genes15111485)
Supplement: Supplementary file 1 [file genes-15-01485-s001.zip › genes-3296466-supplementary.pdf]

Supplementary material

SNP polymorphism is associated with environmental factors in sockeye salmon populations across the Northwest Pacific: insights from redundancy analysis

Genes

Anastasia M. Khrustaleva

Institute of Gene Biology Russian Academy of Sciences (FSBIS IGB RAS), Moscow, Russia

Email: nastia.khrust@gmail.com

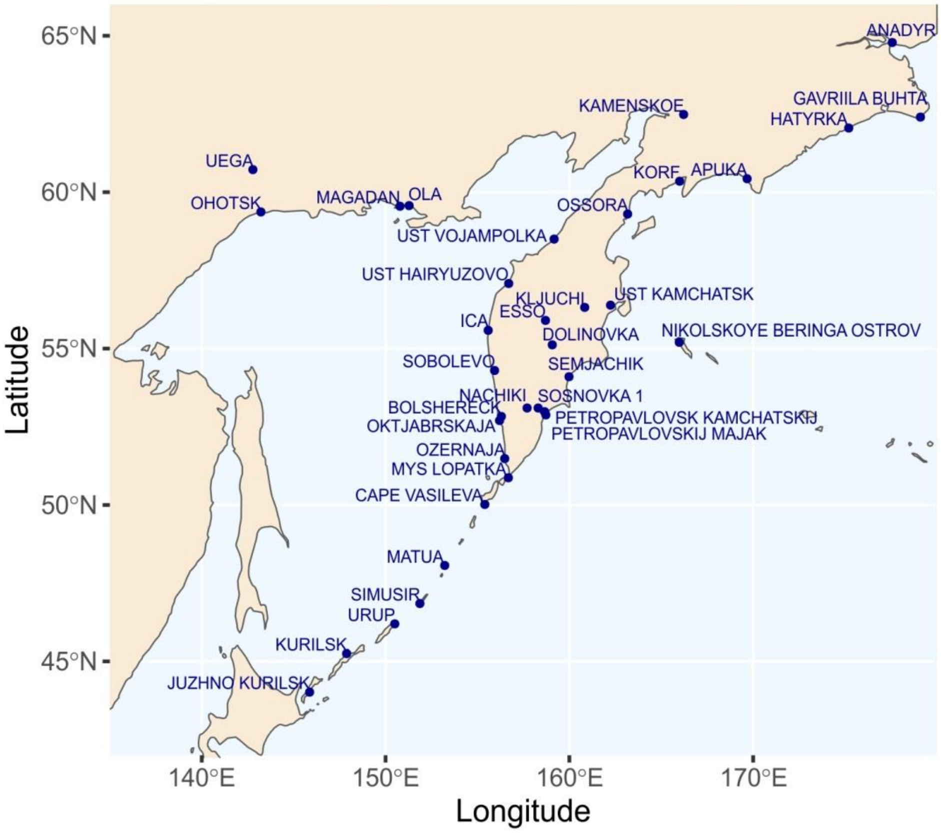

Figure S1. The NOAA weather stations location on the studied area.

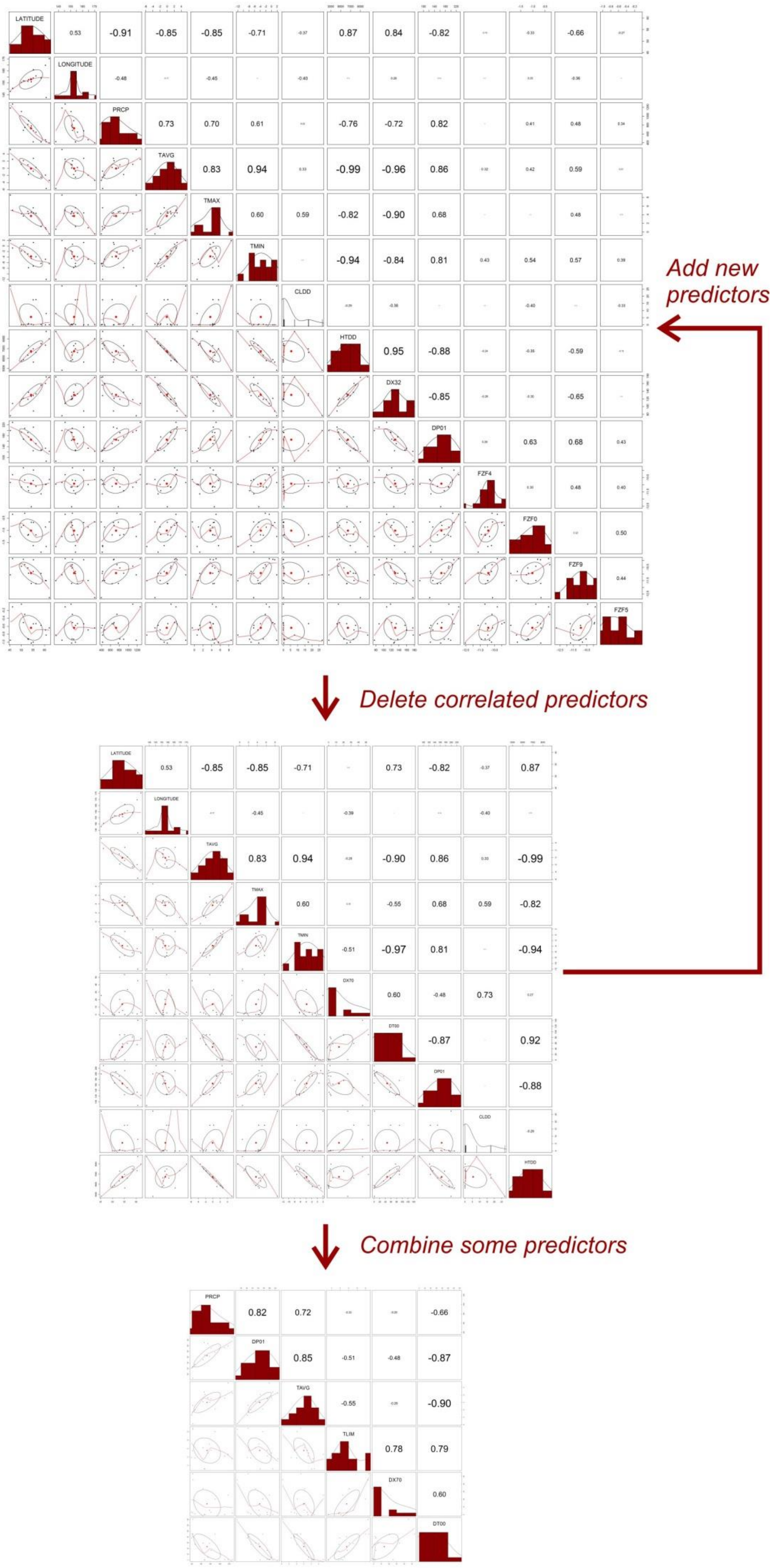

Figure S2. A scheme for predictor selection for the analysis of correlation between environmental factors and allele frequencies of 41 polymorphic SNP loci in sockeye salmon: selection of predictors − removal of predictors with high cross-correlation estimates − addition of new parameters (2-3 iterations) − calculation of derived parameters.

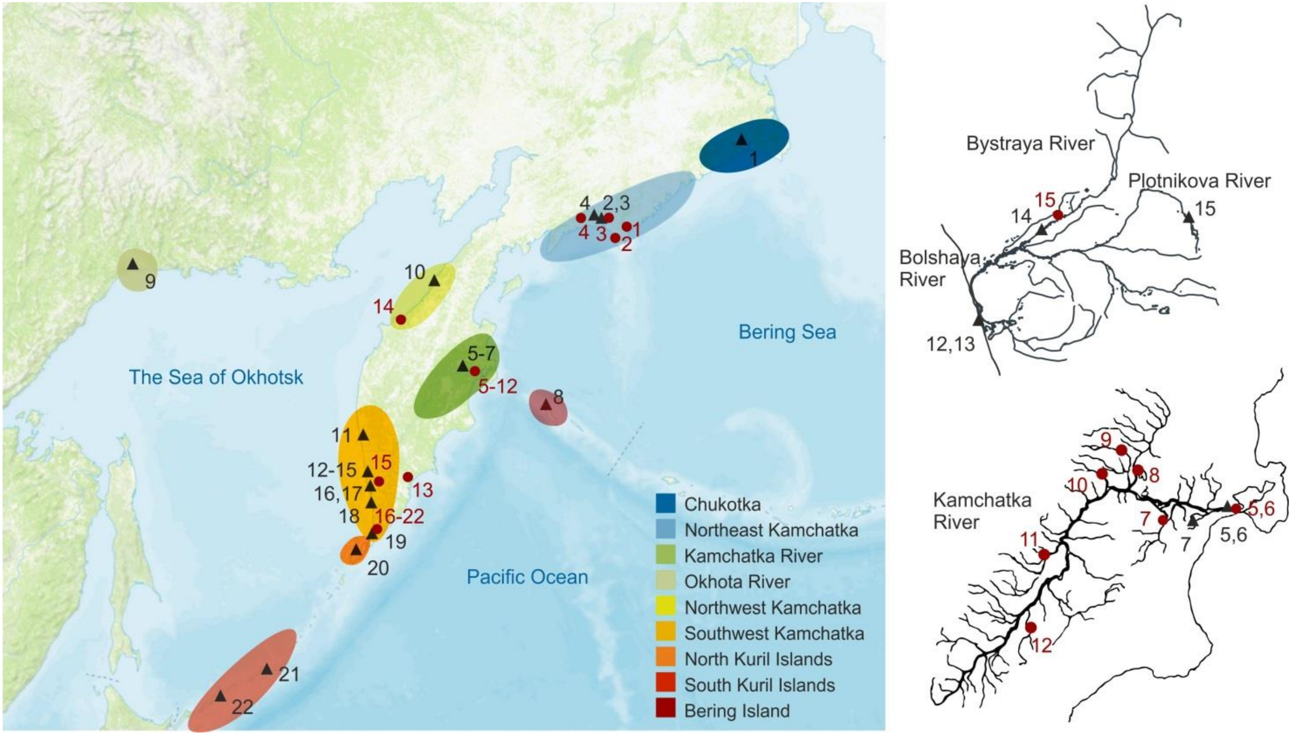

Figure S3. Schematic map of the study area with sampling points (triangles – our collection, red circles – data from Habicht et al.). The point's annotations are given in Table 1 and Supplementary Table S5. The regions listed in Table 1 are shown in different colors.

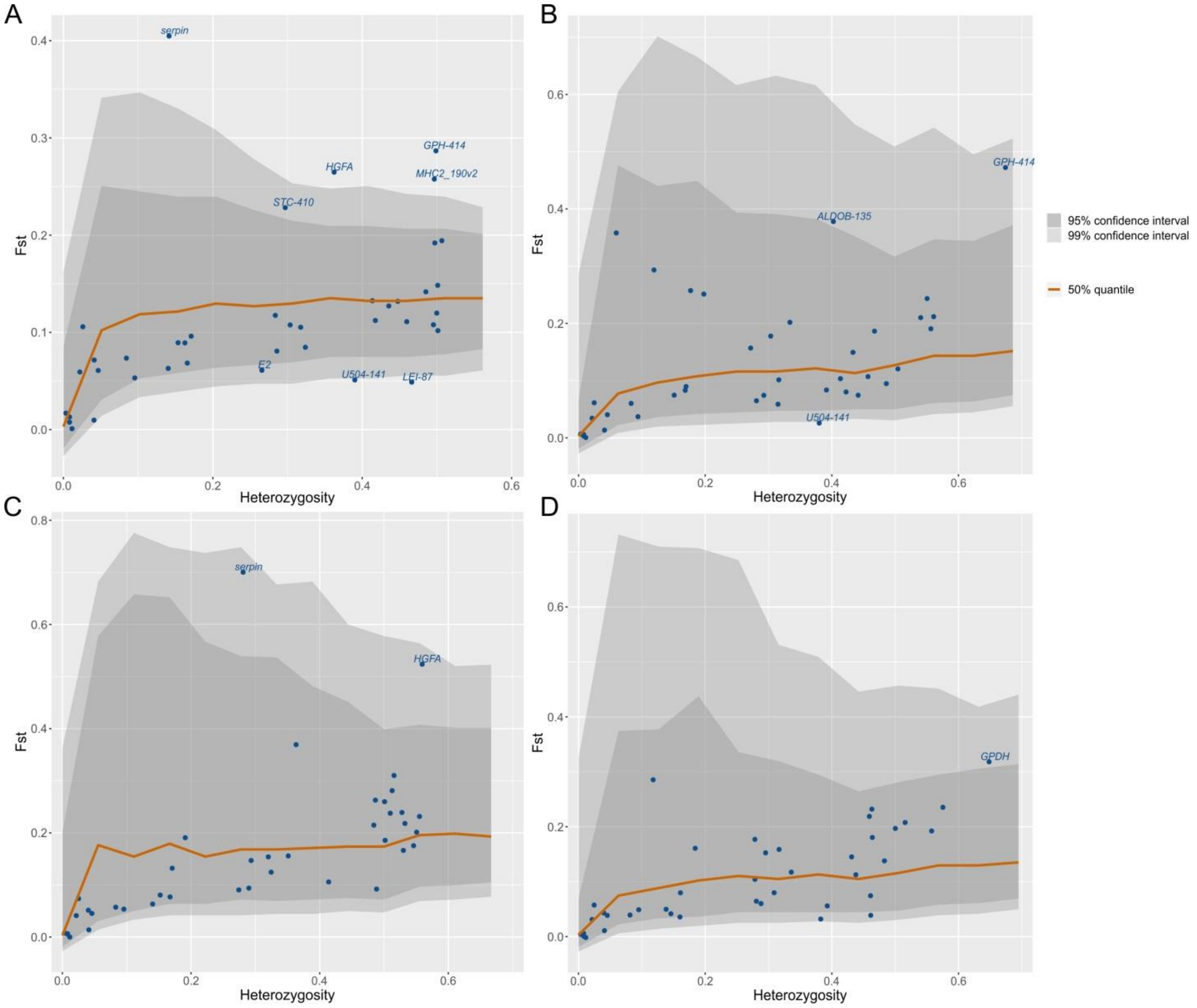

Figure S4. Examples of output images for outlier-SNP detection tests using Arlequin 3.5 for four combinations of samples: (A) – all samples, (B) – all samples vs KP sample, (C) – all continental samples vs all island populations, (D) – all samples vs BS sample. Loci falling above 5% (in the upper part of the graph) and below 1% (in the lower part of the graph) quantile limits were removed as outliers. Here *serpin*, *HGFA*, *GPH-414*, *MHC2_190v2*, *STC-410*, *ALDOB-135*, *GPDH*, and *U504-141 & LEI-87* are considered as outliers.

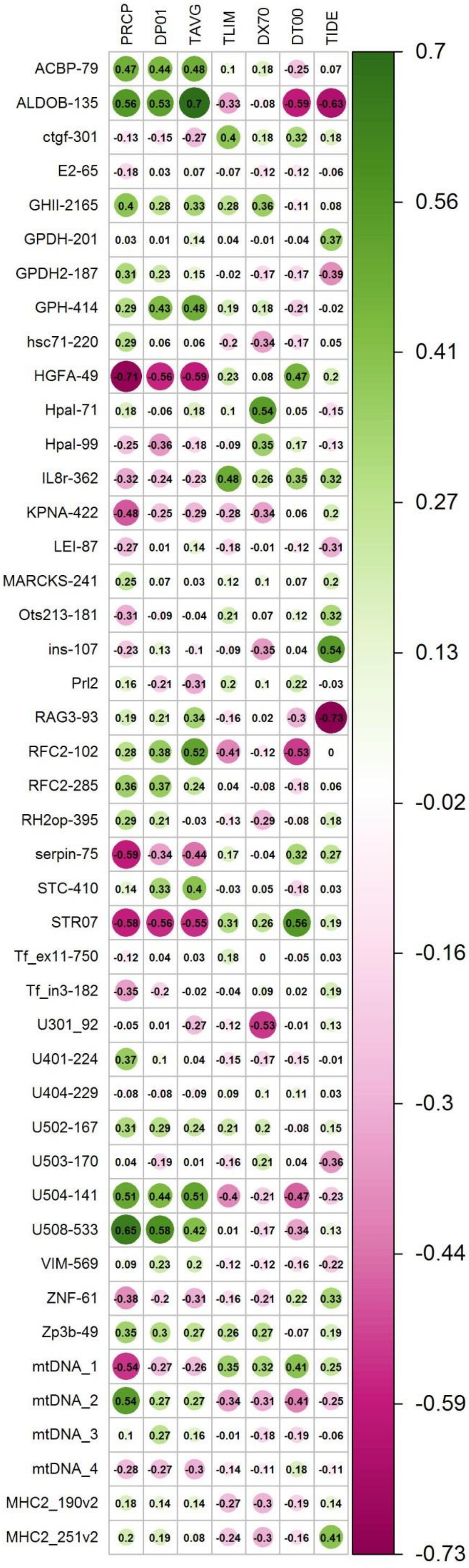

Figure S5. Matrix of the correlation coefficients between allele frequencies of 41 SNPs and environmental indices reflecting abiotic conditions in the reproductive watershed. The numbers in the cells are the correlation coefficients.

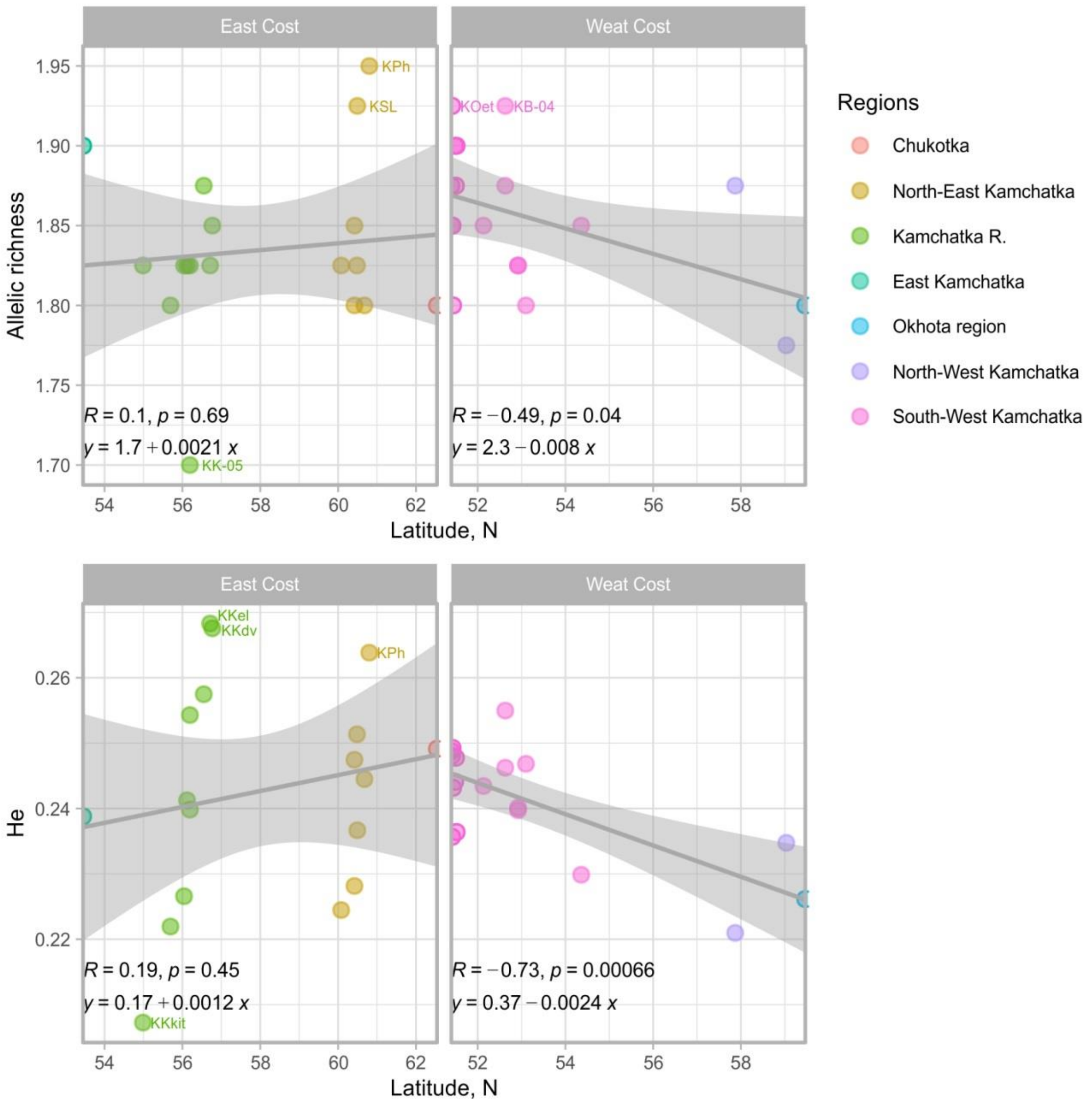

Figure S6. Expected heterozygosity (*He*) and allelic richness (*Ar*) for 41 SNP loci as a function of latitude for sockeye salmon from the Asian coast of the Pacific Ocean: East Coast − East coast of Kamchatka and Chukotka, West Coast − West coast of Kamchatka and the Okhota River. The entire dataset of Habicht et al. (Table S5) was included in the analysis.

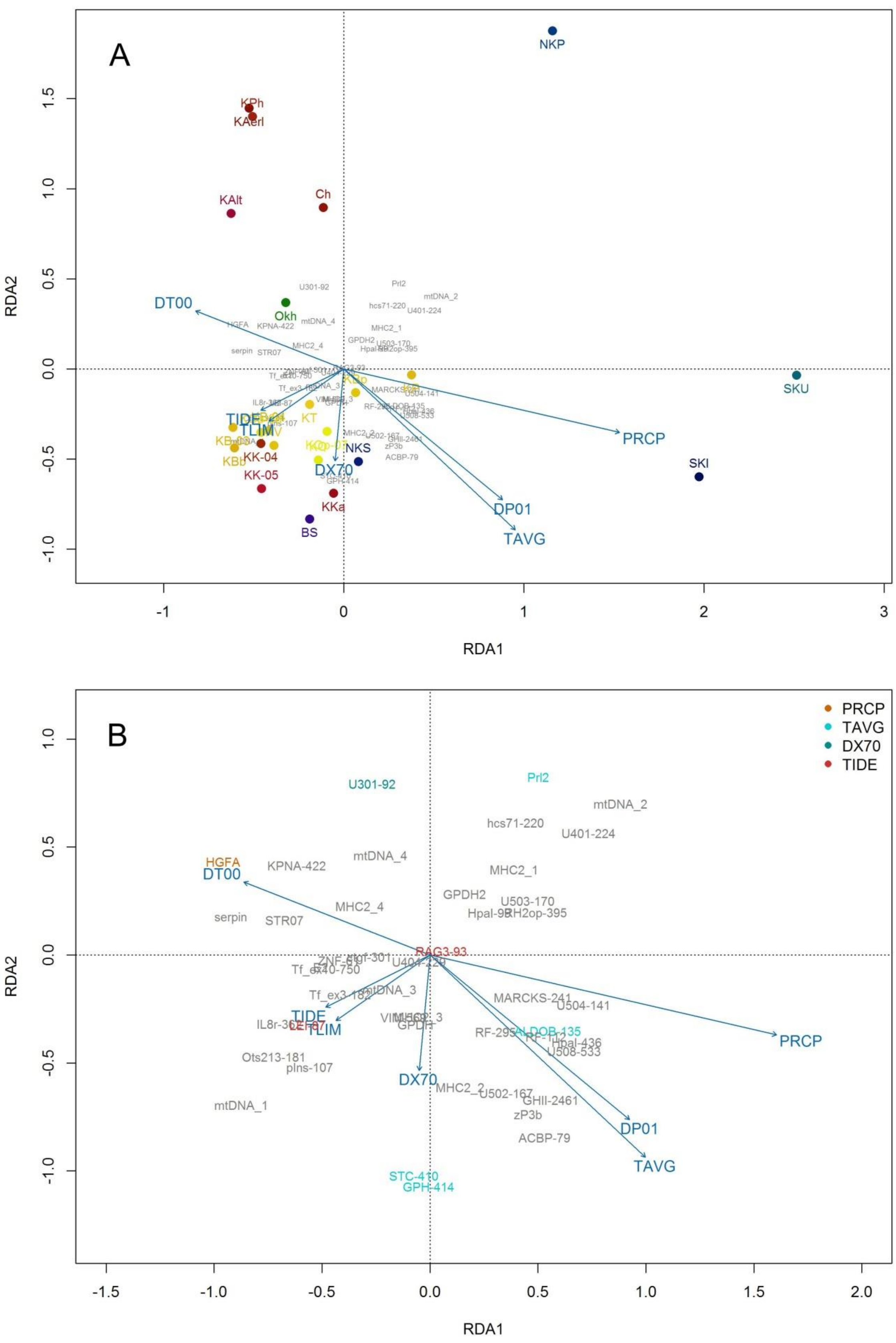

Figure S7. (A) − RDA triplot displaying predictor factors (seven climate and hydrographic indices) (vectors), samples (named points) and dependent variables (SNP loci), and (B) − biplot for genetic traits (B) (loci correlated with the corresponding predictors are marked in corresponding color).

Table S1. Sampling regions and locations, nursery lakes in the watershed, population IDs, and sampling methodology: date and place of catch, coordinates of a river mouth or a lake of catch, fishing gear and collector name if known.

#	Region	Location	Nursery lakes	Pop. ID	Date of catch	Place of catch	Coordinates	Fishing gear	Collector
1	Chukotka, Navarin region	Meinypilgin lake-river system	Pikulneyskoye, High, Middle and Low Vaamochka lakes	Ch	28.07.2004	The creek between the Lower Vaamochka Lake and Vaamochka Lake	62.540115°N, 176.813305°E	fixed nets, seine nets	E.V. Golub (Pacific branch of the VNIRO (“TINRO”))
2	Kamchatka peninsula, Olyutor region	Apuka River, early run	Vatyt-Gythyn Lake	KAerl	24.06.2008-25.06.2008	The lower reaches	60.418785°N, 169.849153°E	seine nets	V.I. Roy (VNIRO)
3		Apuka River, late run	None	KAlt	24.06.2008-25.06.2008	The lower reaches	60.418785°N, 169.849153°E	seine nets	V.I. Roy (VNIRO)
4		Pakhacha River	Potat-Gythyn Lake	KPh	17.06.2005-27.06.2005	The lower reaches	60.799973°N, 169.068806°E	seine nets	E.D. Pavlov (VNIRO)
5	Kamchatka peninsula, East coast	Kamchatka River, late run	Azabachje Lake, Dvu-Yurtochnoye Lake, Kursin Lake, Kamakov lowland lakes	KK-04	29.06.2004-09.07.2004	Downstream, 5 and 30 km from the mouth	56.192783°N, 161.997488°E	seine nets	A.M. Khrustaleva (IGB RAS)
6		Kamchatka River, early run		KK-05	14.06.2005	Downstream, 5 and 30 km from the mouth	56.192783°N, 161.997488°E	seine nets	V.I. Roy (VNIRO)
7		Azabachje Lake, Bushuyka River	Azabachje Lake	KKa	03.07.2004, 13.07.2004	Bushuyka River outfall (tributary of Azabachje Lake)	56.119205°N, 161.854666°E	seine nets	A.M. Khrustaleva (IGB RAS), V.F. Bugaev (Kamchatka branch of the VNIRO (“KamchatNIRO”))
8	Commander Islands	Bering Island, Sarannoye Lake	Sarannoye Lake	BS	7.2008	Sarannoye Lake	55.271646°N, 166.146979°E	seine nets	no data
9	Continental coast of the Sea of Okhotsk	Okhota River	Ueginsky Lakes	Okh	22.07.2004	The lower reaches	59.469398°N, 142.952526°E	seine nets	Magadan branch of the VNIRO (“MagadanNIRO”)
10	Kamchatka peninsula, North-West	Palana River	Plansky Lake	KP	10.07.2003-21.07.2003	Downstream, 10 km from the mouth	59.035209°N, 160.213639°E	gill nets, seine nets	A.M. Khrustaleva (IGB RAS)
11	Kamchatka peninsula, South-West	Bolshaya Vorovskaya River	Vorovsky Lake, No name Lake, Kapovoye Lake	KV	17.07.2007-27.07.2007	The lower reaches	54.359467°N, 156.095038°E	seine nets	Kamchatka branch of the VNIRO (“KamchatNIRO”)
12		Bolshaya River	Nachikinsky Lake, Sokotch Lake	KB-03	23.07.2003-30.07.2003	Downstream, 5-10 km from the mouth	52.628162°N, 156.26196°E	seine nets	A.M. Khrustaleva (IGB RAS)

13		Bolshaya River		KB-04	11.08.2004-20.08.2004	The lower reaches	52.628162°N, 156.26196°E	seine nets	R.A. Zboev, I.N. Kireev (Kamchatka branch of the VNIRO (“KamchatNIRO”))
14		Bolshaya River drainage, Bistraya River	None	KBb	20.07.2004-12.08.2004	The lower course of Bystraya River, 10 km from the Karymay settlement	52.92024°N, 156.609568°E	minnow seine	E.V. Yesin (VNIRO)
15		Bolshaya River drainage, Plotnikova River	Nachikinsky Lake, Sokotch Lake	KBp	09.08.2004-12.082004	Upper reach of Plotnikov River, 10 km from Nachikinskoe Lake	53.10024°N, 157.756895°E	minnow seine	E.V. Yesin (VNIRO)
16		Opala River	Opalinsky Lake	KOp-07	01.07.2007	The lower reaches	52.131653°N, 156.476848°E	seine nets	A.I. Manukhov (VNIRO)
17		Opala River	Opalinsky Lake	KOp-08	17.07.2008-26.08.2009	The lower reaches	52.131653°N, 156.476848°E	seine nets	S.A. Belorusceva (VNIRO)
18		Ozernaya River	Kurilsky Lake, Etamynk Lake	KO	04.08.2003-07.08.2003	The lower reaches	51.484258°N, 156.568087°E	seine nets	A.M. Khrustaleva (IGB RAS)
19	North Kuril Islands	Shumshu Island, Bettobu Lake	Bettobu Lake	NKS	05.08.2008	Ostrognaya Ryver (tributary of Bettobu Lake)	50.751485°N, 156.264268°E	seine nets	K.V. Chudanov (VNIRO)
20		Paramushir Island, Glukhoye Lake	Glukhoye Lake	NKP	07.07.2008-13.07.2008	Shumnaya Ryver (flows into Glukhoye Lake)	50.488928°N, 155.847357°E	seine nets	no data
21	South Kuril Islands	Urup Island, Tokotan Lake	Tokotan Lake	SKU	07.2008-08.2008	Tokotan Lake	45.858739°N, 149.799329°E	seine nets	Sakhalin branch of the VNIRO (“SakhNIRO”)
22		Iturup Island, Krasivoye Lake	Krasivoye Lake	SKI	01.10.2006	Krasivoye Lake	44.624539°N, 147.208965°E	seine nets	Sakhalin branch of the VNIRO (“SakhNIRO”)

Table S2: Characteristics of 45 SNP loci. *He* – mean expected heterosigocity, *Ho* – mean observed heterosigocity, n_a – mean allele count per locus, F_{ST} − fixation index by locus.

#	Locus name	GenBank ID	Putative location	SNP position	Substi-tution	Description	$H_e(SD)$	$H_o(SD)$	$n_a(SD)$	F_{ST}
1	*One_ACBP-79*	DQ386287	acyl-coenzyme A-binding protein (ACBP) gene	79	A/G	mRNA	0.39(0.14)	0.36(0.13)	1.91(0.29)	0.132
2	*One_ALDOB-135*	DQ386280	aldolase B (ALDOB) gene, partial sequence	135	G/A	noncoding region	0.22(0.15)	0.21(0.13)	1.86(0.35)	0.117
3	*One_CO1*	AY353070	haplotype TAGG DNA cytochrome oxidase I-like gene, partial sequence; mitochondrial	7061	T/C	synonymous	haploid		1.96(0.2)	−
4	*One_ctgf-301*	DQ386288	connective tissue growth factor (CTGF) gene	287	G/T	mRNA	0.01(0.02)	0.01(0.02)	1.23(0.43)	0.013
5	*One_Cytb_17*	AY353063	GT DNA cytochrome b-like gene, partial sequence; mitochondrial	16162	G/A	synonymous	haploid		1.17(0.38)	−
6	*One_Cytb_26*	AY353064	GC DNA cytochrome b-like gene, partial sequence; mitochondrial	16168	T/C	synonymous	haploid		1.96(0.2)	−
7	*One_E2*	DQ025695	One.E2.31.36 genomic sequence similar to type II keratin E2	65	A/G	mRNA	0.25(0.11)	0.26(0.14)	2(0)	0.061
8	*One_GHII-2461*	U14535.1	type-2 growth hormone gene, complete cds	43	T/A	intron	0.14(0.14)	0.15(0.15)	1.82(0.39)	0.089
9	*One_GPDH*	DQ025723	One.GPDH.40.61 genomic sequence similar to glycerol-3-phosphate dehydrogenase (GPDH)	187	C/G	mRNA	0.44(0.11)	0.44(0.14)	2(0)	0.108
10	*One_GPDH2*	DQ025724	One.GPDH.56.48 genomic sequence similar to glycerol-3-phosphate dehydrogenase (GPDH)	201	C/T	mRNA	0.13(0.11)	0.12(0.12)	1.95(0.21)	0.063
11	*One_GPH-414*	DQ386289	glycoprotein hormone alpha-subunit (GPH) gene	414	T/C	intron	0.36(0.13)	0.35(0.14)	2(0)	0.287
12	*One_hcs71-220*	DQ386293	major heat shock protein-like protein (HSC71) gene, partial sequence	220	A/C	intron	0.4(0.13)	0.39(0.13)	2(0)	0.111
13	*One_HGFA*	DQ025719	One.HGFA.22.46 genomic sequence similar to hepatocyte growth factor activator/GRAAL (HGFA)	49	A/T	unknown	0.26(0.14)	0.24(0.14)	1.95(0.21)	0.265
14	*One_HpaI-436*	DQ386294	One-436 HpaI repeat element-like sequence similar to HpaI repeat element	79	A/T	unknown	0.42(0.08)	0.39(0.09)	2(0)	0.148
15	*One_HpaI-99*	DQ386281	clone One-99 HpaI repeat element-like sequence	99	C/T	unknown	0.08(0.1)	0.08(0.1)	1.5(0.51)	0.073
16	*One_IL8r-362*	FM206384, GU570948.1	Oncorhynchus mykiss partial il-8 gene for interleukin 8, promoter region (interleukin-8 receptors), isolate 98JN-020 chemokine receptor (IL8R) gene	362	C/T	promoter region	0.29(0.1)	0.27(0.13)	2(0)	0.108
17	*One_KPNA-422*	DQ386282	karyopherin alpha 2 (KPNA2) gene	422	A/G	unknown	0.34(0.14)	0.34(0.14)	1.95(0.21)	0.133
18	*One_LEI-87*	DQ386279	leukocyte elastase inhibitor (LEI) gene	87	A/G	unknown	0.45(0.11)	0.47(0.13)	2(0)	0.049
19	*One_MARCKS-241*	GU570949.1	myristoylated alanine-rich protein kinase (MARCKS) gene	241	A/T	unknown	0.01(0.01)	0.01(0.01)	1.36(0.49)	0.001
20	*One_MHC2_190v2*	AY386256	major histocompatibility complex class II B1 (Onne-DAB) gene, Onne-DAB-3 allele, partial cds	190	T/G	exon	0.37(0.13)	0.33(0.16)	1.97(0.12)	0.258

21	*One_MHC2_251v2*	AY386257	major histocompatibility complex class II B1 (Onne-DAB) gene, Onne-DAB-4 allele, partial cds	251	C/T	intron	0.41(0.12)	0.35(0.17)	1.98(0.1)	0.192
22	*One_Ots213-181*	DQ386285	clone Ots213 genomic sequence	220	T/G	unknown	0.16(0.15)	0.15(0.14)	1.86(0.35)	0.096
23	*One_p53-576*	DQ386284	p53 tumour suppression gene, partial sequence	534	A/C	unknown	monomorphic		1(0)	–
24	*One_pIns-107*	DQ025686	One.ins.10.30 genomic sequence similar to insulin gene	107	C/T	unknown	0.46(0.06)	0.43(0.08)	2(0)	0.102
25	*One_Prl2*	AY353071	haplotype G prolactin II gene, partial cds (coding sequence)	187	G/T	coding	0.44(0.1)	0.44(0.13)	2(0)	0.12
26	*One_RAG1-103*	DQ386290	recombination activating protein (RAG1) gene	103	A/T	unknown	monomorphic		1(0)	–
27	*One_RAG3-93*	DQ386291	recombination activating protein (RAG1) gene	93	C/T	mRNA	0.03(0.06)	0.03(0.05)	1.5(0.51)	0.072
28	*One_RF-112*	AB435387	12-RFa mRNA for 12-RF amide peptide, complete cds	112	A/G	exon, synonymous	0.26(0.13)	0.26(0.13)	2(0)	0.081
29	*One_RF-295*	AB435387	12-RFa mRNA for 12-RF amide peptide, complete cds	259	A/T	exon	0.04(0.08)	0.04(0.08)	1.5(0.51)	0.061
30	*One_RH2op-395*	DQ386277	RH2 opsin (RH2op) gene, partial sequence	395	T/G	unknown	0.04(0.04)	0.04(0.04)	1.73(0.46)	0.01
31	*One_serpin*	DQ025707	clone One.serpin.50.38 genomic sequence similar to SERine proteinase Inhibitors (serpin)	75	T/G	unknown	0.09(0.13)	0.09(0.14)	1.73(0.46)	0.405
32	*One_STC-410*	DQ386278	stanniocalicin (STC) gene	410	C/T	intron	0.21(0.16)	0.2(0.16)	1.95(0.21)	0.228
33	*One_STR07*	DQ386286	clone STR07 genomic sequence	182	C/G	unknown	0.29(0.17)	0.27(0.16)	1.91(0.29)	0.085
34	*One_Tf_ex10-750*	AH015399.2	transferrin (Tf) gene	750	A/G	exon	0.42(0.13)	0.39(0.15)	2(0)	0.142
35	*One_Tf_ex3-182*	AH015399.2	transferrin (Tf) gene	182	A/G	intron	0.01(0.02)	0(0.01)	1.23(0.43)	0.008
36	*One_U301-92*	DQ267490	FK506-binding protein 12-like (FKBP12) gene	92	G/T	unknown	0.15(0.13)	0.14(0.14)	1.91(0.29)	0.068
37	*One_U401-224*	GU570950.1	unknown	224	A/C	unknown	0.36(0.1)	0.34(0.1)	2(0)	0.112
38	*One_U404-229*	GU570951.1	unknown	229	C/T	unknown	0.03(0.07)	0.03(0.07)	1.27(0.46)	0.106
39	*One_U502-167*	GU570952.1	unknown	167	A/G	unknown	0(0.02)	0(0.02)	1.09(0.29)	0.017
40	*One_U503-170*	GU570953.1	unknown	170	G/T	unknown	0.28(0.16)	0.28(0.17)	1.91(0.29)	0.105
41	*One_U504-141*	GU570954.1	unknown	141	A/C	unknown	0.36(0.14)	0.36(0.14)	1.95(0.21)	0.051
42	*One_U508-533*	GU570955.1	unknown	162	C/T	unknown	0.1(0.1)	0.08(0.09)	1.77(0.43)	0.053
43	*One_VIM-569*	DQ386292	vimentin (VIM) gene	563	A/G	unknown	0.14(0.13)	0.15(0.14)	1.91(0.29)	0.089
44	*One_ZNF-61*	BT057144	ER lumen protein-retaining receptor 2	61	C/A	mRNA	0.37(0.13)	0.34(0.13)	2(0)	0.127
45	*One_zP3b*	DQ025739	Oncorhynchus mykiss C-C chemokine receptor type 9-like	49	A/C	mRNA	0.03(0.06)	0.03(0.07)	1.27(0.46)	0.059

Table S3. The NOAA weather stations data used in this study, weather stations location, years of observation, number of observations, and corresponding spawning lake-river systems.

#	Station Name	Station ID	Years	*n*	Elevation	Latitude	Longitude	Lake-River Systems	Pop ID
1	Hatyrka	RSM00025767	1990-2011	15	11	62.05	175.2	Vaamochka Lake	Ch
2	Apuka	RSM00025956	1990-2010	28	3	60.43	169.67	Apuka River, early run	KAerl
3	Apuka	RSM00025956	1990-2010	28	3	60.43	169.67	Apuka River, late run	KAlt
4	Apuka	RSM00025956	1990-2010	28	3	60.43	169.67	Pakhacha River	KPh
5	Dolinovka	RSM00032447	1990-2010	20	101	55.12	159.07	Kamchatka River, late run	KK-04
6	Kljuchi	RSM00032389	1990-2010	21	28	56.3167	160.8331	Kamchatka River, early run	KK-05
7	Kljuchi	RSM00032389	1990-2010	21	28	56.3167	160.8331	Kamchatka River drainage, Azabachje Lake, Bushuyka River	KKa
8	Nikolskoye Beringa Ostrov	RSM00032618	1990-2010	19	16	55.2	165.98	Bering Island, Sarannoye Lake	BS
9	Uega	RSM00024982	1990-2010	20	396	60.72	142.78	Okhota River	Okh
10	Predicted by a linear approximation					59.08	159.84	Palana River	KP
11	Predicted by a linear approximation					57.99	158.3	Tigil River	KT
12	Sobolevo	RSM00032477	1990-2010	19	25	54.3	155.93	Bolshaya Vorovskaya River	KV
13	Bolshereck	RSM00032562	1990-2010	20	30	52.83	156.3	Bolshaya River	KB-03
14	Bolshereck	RSM00032562	1990-2010	20	30	52.83	156.3	Bolshaya River	KB-04
15	Bolshereck	RSM00032562	1990-2010	20	30	52.83	156.3	Bolshaya River drainage, Bistraya River	KBb
16	Nachiki	RSM00032539	1990-2010	19	317	53.1	157.7	Bolshaya River drainage, Plotnikova River	KBp
17	Predicted by a linear approximation					51.98	156.483	Opala River	KOp-07
18	Predicted by a linear approximation					51.98	156.483	Opala River	KOp-08
19	Ozernaja	RSM00032594	1950-1992	37	28	51.483	156.483	Ozernaya River	KO
20	Mys Lopatka	RSM00032213	1990-2007	14	48	50.87	156.68	Shumshu Island, Bettobu Lake	NKS
21	Cape Vasileva	RSM00032217	1951-1992	33	11	50.017	155.4	Paramushir Island, Glukhoye Lake	NKP
22	Urup	RSM00032186	1960-1993	28	76	46.2	150.5	Urup Island, Tokotan Lake	SKU
23	Kurilsk	RSM00032174	1990-2010	21	25	45.25	147.88	Iturup Island, Krasivoye Lake	SKI

Table S4. The environmental factors used in the present study.

#	Factor ID	Factor	Interpretation	Units
1	TAVG	Average Temperature	the average annual air temperature	°C
2	TLIM	Temperature variation range (Maximum temperature minus Minimum temperature (TMAX-TMIN))	the value of the annual air temperature variation range	°C
3	DT00	Number days with minimum temperature less than or equal to 0.00°F	the duration of cold period	days
4	DX70	Number days with maximum temperature > 70°F (21.1°C)	the duration of warm period	days
5	PRCP	Precipitation	the average annual precipitation amount	mm
6	DP01	Number of days with greater than or equal to 0.1 inch of precipitation	the number of rainy/snowy days per year	days
7	TIDE	Height of the maximal tide	the height of the maximal tide in the estuary	m

Table S5. Samples characteristics, regions and locations, population IDs, date of catch, coordinates, and summary statistics for 40 SNP loci according to the data from (Habicht et al., 2010): mean expected (*He*) heterozygosities, allelic richness (*Ar*).

#	Region	Location	Pop. ID	Date of catch	Coordinates	*n*	*Ar(SD)*	*He(SD)*
1	Kamchatka peninsula, Olyutor region	Severnaya Lagoon	KSL	6/26/2002	60.5°N, 170.62°E	98	1.93(0.33)	0.21(0.18)
2		Anana Lagoon	KAL	6/24/2002	60.04°N, 170.22°E	80	1.78(0.42)	0.21(0.19)
3		Apuka River, Vatit Lake	KAvat	8/7/2002	60.67°N, 170.25°E	51	1.78(0.42)	0.21(0.18)
4		Pakhacha River, Potat Lake	KPhpot	7/29/2001	60.67°N, 167.6°E	50	1.74(0.43)	0.19(0.18)
5	Kamchatka River basin	Kamchatka River, late run	KKlt-98	7/21/1998	56.23°N, 162.5°E	100	1.78(0.42)	0.21(0.19)
6		Kamchatka River, early run	KKerl-98	6/1/1998	56.23°N, 162.5°E	78	1.74(0.44)	0.19(0.17)
7		Hapiza River	KKhap	9/2/1998	56.2°N, 161.25°E	146	1.74(0.44)	0.23(0.19)
8		Elovka River	KKel	1995	56.58°N, 160.75°E	109	1.78(0.42)	0.25(0.2)
9		Dvu 'Yurta River	KKdv	1995	56.55°N, 160.13°E	88	1.78(0.43)	0.25(0.21)
10		Belaya River	KKbel	1995	56.43°N, 160.35°E	81	1.85(0.37)	0.23(0.19)
11		Kozireuka River	KKkoz	1994	56.03°N, 159.78°E	40	1.74(0.44)	0.22(0.18)
12		Kitilgina River, early run	KKkit	6/29/1998	55.07°N, 159.13°E	28	1.78(0.42)	0.2(0.18)
13	Kamchatka peninsula, South-East	Avacha Bay	KAv	2002	53.03°N, 158.44°E	60	1.85(0.35)	0.21(0.18)
14	Kamchatka peninsula, North-West	Tigil River	KT	6/18/2002	58°N, 158.27°E	107	1.81(0.41)	0.17(0.18)
15	Kamchatka peninsula, South-West	Bistraya River	KBb-98	8/16/1998	53.35°N, 157.47°E	56	1.74(0.44)	0.2(0.19)
16	Ozernaya River basin, Kuril Lake	R. Vychenkia	KOvych	7/28/2000	51.48°N, 156.57°E	96	1.9 (0.3)	0.236 (0.187)
17		Bay Northern Far	KOsev	8/26/2002	51.48°N, 156.57°E	50	1.88 (0.33)	0.248 (0.19)
18		Bay Oladochnaya	KOol	10/8/2000	51.48°N, 156.57°E	50	1.8 (0.41)	0.243 (0.191)
19		Bay Gavryushka	KOgav	8/25/2002	51.48°N, 156.57°E	50	1.85 (0.36)	0.249 (0.189)
20		R. Kirushutk	KOkir	7/31/2000	51.48°N, 156.57°E	49	1.88 (0.33)	0.249 (0.198)
21		Bay Khakytsin	KOhak	8/31/2002	51.48°N, 156.57°E	50	1.85 (0.36)	0.236 (0.188)
22		R. Etamynk	KOet	8/12/2002, 8/21/1990, 9/28/1990	51.48°N, 156.57°E	127	1.93 (0.27)	0.248 (0.194)

Table S6. Correlation coefficients of allele frequencies of SNP loci with the latitude of the spawning watersheds for which the correlation was significant in at least one test (*** − $p < 0.001$, ** − $p < 0.01$, * − $p < 0.05$) along the all Asian coast of the North Pacific (All), coast of Chukotka & East Kamchatka (East Cost), and the Continental coast of the Sea of Okhotsk & West Kamchatka (West Coast).

Locus	All	West Coast	East Coast
ACBP-79	−0.66***	−0.28	−0.8*
ALDOB-135	−0.57**	−0.8**	−0.73*
GHII-2461	−0.46*	0.22	−0.72*
GPH-414	−0.43*	−0.74*	−0.84**
hcs71-220	−0.11	−0.32	0.85**
HGFA	0.72***	0.11	0.43
HpaI-436	−0.23	0.8**	−0.76*
mtDNA_1	0.39	0.45	−0.79*
Prl2	0.07	0.84**	0.63
RAG3-93	−0.2	−0.72*	−0.07
RF-112	−0.37	−0.27	−0.86**
RH2op-395	−0.11	0.71*	0.36
STC-410	−0.3	−0.74*	−0.67
Tf_ex10-750	0.03	−0.71*	−0.39
U503-170	−0.03	0.91***	0.37
ZNF-61	0.4	0.74*	0.46
